# The Correlation between Metal Mixed Exposure and Lung Function in Different Ages of the Population

**DOI:** 10.3390/metabo14030139

**Published:** 2024-02-26

**Authors:** Zhongwen Chen, Huiwen Gu, Ruiqi Zhou, Shuqun Cheng

**Affiliations:** Department of Occupational and Environmental Health, College of Public Health, Chongqing Medical University, Chongqing 400016, China; 2021120755@stu.cqmu.edu.cn (Z.C.); guhuiwen@stu.cqmu.edu.cn (H.G.); 2022110612@stu.cqmu.edu.cn (R.Z.)

**Keywords:** metals, GLM, Qgcomp, BKMR, pulmonary function

## Abstract

Herein, we explored the overall association between metal mixtures and lung functions in populations of varying ages and the relationship among the associated components. The 2007–2012 National Health and Nutrition Examination Survey data of 4382 American participants was analyzed, and generalized linear, elastic net, quantile g–computation, and Bayesian kernel machine regression models were used to evaluate the relationship between exposure to the metal mixture and lung function at various ages. The results of barium exposure at distinct stages revealed that children and adolescents exhibited greater lung function changes than those in adults and the elderly. Additionally, compared with children and adolescents, cadmium– and arsenic–containing metabolites contributed to nonconductive lung function changes in adults and the elderly exposed to metal mixtures. The results showed that the effects of exposure to metal mixtures on lung function in children and adolescents were predominantly caused by lead and barium. Altogether, children and adolescents were found to be more susceptible to metal–exposure–mediated lung function changes than adults and the elderly.

## 1. Introduction

Metals are abundant in nature and can threaten human well-being through various routes including drinking water, diet, air inhalation, and skin contact [1,2,3]. Awareness regarding the negative effects of metals on human health has been increasing over time. Metals can accumulate in the human tissues, organs such as kidneys, skeletal system, nervous system, and cardiovascular system over long periods, contributing to damage to the corresponding organs and systems [4,5]. Despite the legislation and regulations to mitigate metal pollution and related human exposure, metals persist within the environment, creating difficulties in their eradication [5,6]. Consequently, environmental metal pollutants remain significant ecological and health concerns worldwide, warranting further studies [7].

Lung function is a crucial screening parameter for evaluating lung injury, as an impaired lung function is a prominent characteristic of lung disease, and its severity can be assessed utilizing such parameters [8]. Additionally, lung function may be used as a diagnostic parameter for lung issues such as chronic obstructive pulmonary disease (COPD). The mortality rate associated with chronic respiratory illnesses in the United States has increased by approximately 30% from 1980 to 2014 [9]; hence, comprehending the underlying causes of early lung function decline is crucial for reducing lung disease-associated morbidity and mortality [10].

Reportedly, arsenic and tungsten exposure in certain environments can contribute to the development of chronic lung diseases [11]. Furthermore, although trace metals such as selenium and molybdenum are essential for the human body, excessive levels of these elements can result in lung diseases [12,13,14]. Previous studies have primarily investigated the relationships between individual metal exposure and lung function; however, in real–world scenarios, humans are often exposed to multiple metals simultaneously [15]. Moreover, the interactions among these metals may amplify or diminish the toxicity or beneficial effects of individual metals [16,17]. Contemporary studies investigating the correlation between exposure to an individual metal and lung function possess certain limitations and inadequacies. Moreover, studies on the effects of exposure to multiple metals on lung function are limited, necessitating further investigations.

Compared with adults, the respiratory tracts of children are more complex, with unique anatomical and physiological characteristics [18,19]. Reportedly, acute airway obstruction is prevalent among infants and children [20]. Interstitial lung disease is widely spread in children and adolescents, and pulmonary fibrosis in adults [21]. Additionally, the anatomy, physiology, and immunity of the respiratory system evolve with increasing age [22], whereas respiratory muscle strength decreases with age, which may contribute to the inability of the body to remove harmful substances [23]. Respiratory structure varies among the population at distinct stages and is divergent, resulting in varying sensitivity to external factors. Present studies focus on the effect of single–metal exposure on lung function in a population at a certain stage, and none have determined the effect of metal exposure on lung function in populations at varying stages. Therefore, herein, we examined the effects of mixed metal exposure on lung function in children and adolescents (6–18 years), adults (19–59 years), and the elderly (≥60 years) to analyze the effects of metal exposure on lung function among these distinct stages of the population and prevent metal exposure in a targeted manner.

Additionally, existing studies have predominantly investigated the association between individual metal exposure and lung function, with limited studies on the effects of multi–metal exposure on respiratory function. To address this knowledge gap, we utilized data from the National Health and Nutrition Examination Survey (NHANES) to evaluate the relationship between multi–metal exposure and lung function using the generalized linear model (GLM), elastic net (ENET) model, quantile g–computation (Qgcomp) model, and Bayesian kernel machine regression (BKMR) model. The findings of this study may propose research concepts regarding the consequences of multi–metal exposure on lung function, along with strategies for its prevention and treatment.

## 2. Materials and Methods

### 2.1. Study Design and Participants

NHANES is a nationally representative cross-sectional survey conducted by the United States Centers for Disease Control and Prevention to comprehensively assess the health and nutritional status of the general population in the United States. Written informed consent was obtained from all study participants before the survey. Details of the sampling procedure, design, and associated data involved in this study are available on the NHANES official website.

**Figure 1:**
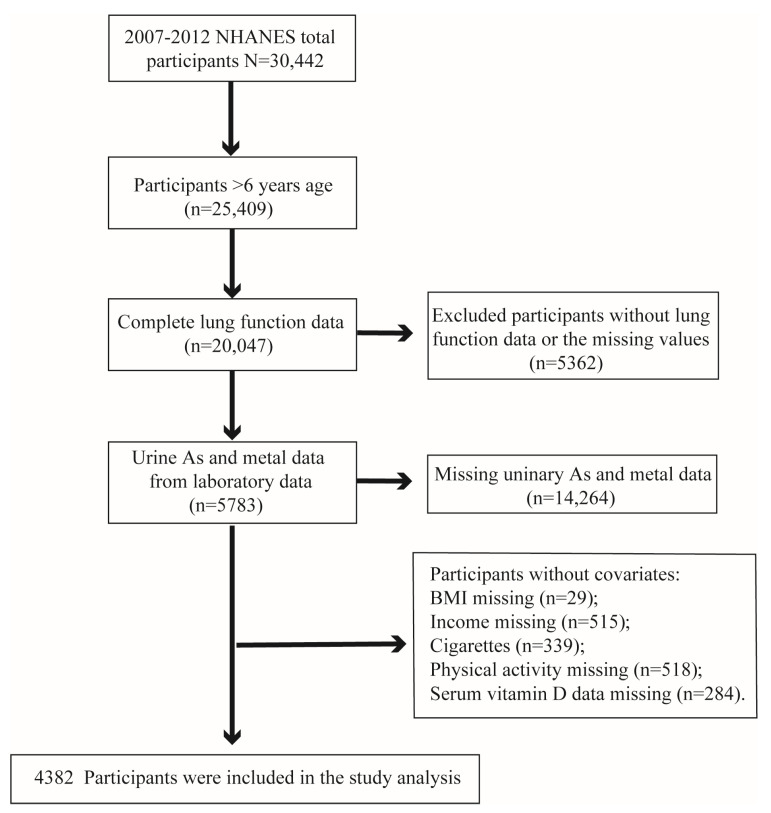
Flowchart depicting the inclusion of subjects in our study, NHANES, USA, 2007–2012.

Herein, we used data obtained in 2007–2018, 2009–2010, and 2011–2012 (three cycles) from the NHANES official website; a total of 30,442 respondents were surveyed from 2007 to 2012. Following the inclusion and exclusion criteria of similar studies to reduce the selection bias [24,25], first, 5033 participants who did not perform laboratory tests were excluded, followed by those who lacked lung function measurements or had missing or unreliable values (*n* = 5362). Additionally, participants with incomplete laboratory tests, including urinary metals and urinary As, were excluded from the remaining sample (*n* = 14,264). An additional 1401 individuals who lacked basic covariate data, such as family income and poverty ratio, race, education, marital status, body mass index (BMI), physical activity, cigarette consumption, and alcohol consumption were excluded to ensure the accuracy of the results. The selection process of participants is shown in Figure 1.

### 2.2. Measurement of Vital Capacity

Detailed information regarding vital capacity measurements can be found on the NHANES official website [26]. Herein, mainly forced expiratory volume in one second (FEV1), forced vital capacity (FVC), peak expiratory flow (PEF), forced expiratory flow between 25% and 75% FVC (FEF25–75%), and baseline forced expiratory time (FET) were analyzed.

### 2.3. Environmental Chemicals

The following metals were examined in this study: arsenic (As), arsenobetaine (AsB), dimethylarsinic acid (DMA), barium (Ba), cadmium (Cd), cobalt (Co), cesium (Cs), molybdenum (Mo), lead (Pb), antimony (Sb), thallium (Tl), tungsten (W), uranium (U), and mercury (Hg). To address values below the detection limit, the NHANES laboratory procedure was followed, that is, replacing low values with the detection limit divided by the square root of two. Detailed analysis information and limit of detection (LOD) values for each chemical are provided in the laboratory method documentation available on the NHANES official website [27,28].

### 2.4. Covariates

Consistent with previous similar studies [25,29,30], demographic data collection involved categorizing participants into specific age groups: children and adolescents (6–18 years), adults (19–59 years), and the elderly (≥60 years) [31]. Sex was recorded as male or female; race was categorized as non-Hispanic White, non–Hispanic Black, Mexican American, other Hispanic, and other races; and family income and poverty ratio (PIR) were categorized as <1.30, 1.30–3.50, and >3.50 [28]. Body measurements, including BMI, were recorded by trained health technicians and further categorized into underweight (<18.5 kg/m^2^), normal (18.5–24.9 kg/m^2^), overweight (25–29.9 kg/m^2^), and obese (≥30.0 kg/m^2^). Serum cotinine levels reflect the markers of tobacco exposure in vivo [25].

### 2.5. Statistical Analyses

NHANES uses a multi–stage complex design, using WTSA2YR, which ensures that the data are representative, and the data analyses follow the guidelines provided on the NHANES official website. The weighted descriptive statistics were expressed as numbers (N) and percentages (%), the continuous variables were expressed as weighted averages and standard deviations, and the skewed variables were expressed as median and quartile ranges. The *t*-test was used for the normal distribution data, the Mann–Whitney U test was used for skewed distribution data, and the chi–square test was used for categorical data. To elucidate the effects of urine dilution on the measurement results, the relationship between metal exposure levels and lung function was analyzed using urinary creatinine-corrected urinary metal concentration (μg/g) [32]. The Pearson correlation coefficient was utilized to assess the correlation among the concentrations of urinary metals.

#### 2.5.1. ENET Model and GLM

ENET is a regularization model based on Ridge regression and least absolute shrinkage and selection operator regression [33,34]. When multiple highly correlated variables are related to the outcome, two or more are selected by ENET, demonstrating a certain stability for the selected variables [35]. Owing to the presence of covariance among the metals, herein, we utilized the ENET model to screen for elements in the highly correlated metals in association with the dependent variable, namely, lung function, and calculated the corresponding beta coefficients (β) to quantify the relationship between urinary metal concentrations and lung function. Furthermore, the selected confounding covariates were included in the ENET regression model as unpunished variables, where the further away the value was from 0, the stronger the relationship between the metal element and lung function.

A GLM was utilized to assess the effect of both single- and multi-metal exposure on lung function. The coefficient “b” was utilized to quantify the effect magnitude. Herein, the following two models were developed: Model 1, a rough model, and Model 2, which was adjusted for various factors including age, gender, race, education level, PIR, BMI, physical activity, and serum cotinine levels. Additionally, both models were adjusted for urinary creatinine. If the regression *p*-value of the metal as a continuous variable was <0.05, the difference was considered statistically significant.

#### 2.5.2. Qgcomp Model

Considering the easy concurrent exposure of various heavy metals to people, different metals may be highly correlated. The recently developed Qgcomp model was used to estimate the overall associations between the metal mixtures and lung function. This novel statistical strategy presents the advantages of the simplicity of weighted quantile sum regression (WQS) model reasoning and the adaptability of g calculation, and it can evaluate the cumulative effects of multiple chemicals in different directions [36]. However, a limitation of WQS regression is that it assumes all associations are in the same direction. In contrast to WQS, Qgcomp does not require the association among all heavy metals and lung function levels in the same direction [37]. When exposure effects are nonlinear and nonadditive, the WQS model yields biased estimates of the quadratic exposure effects, whereas Qgcomp yields unbiased estimates of the exposure effects and associated variances. Therefore, Qgcomp can provide a more realistic estimate of the effect of the mixture as a whole and more precise estimates. The Qgcomp regression model was adjusted for age, gender, race, education level, PIR, BMI, physical activity, and serum cotinine levels.

#### 2.5.3. BKMR Model

The BKMR model was used to further explore the dose–response relationship between urinary metals and lung function, along with the interactions among urinary metals. BKMR is widely used in epidemiological studies involving mixed environmental exposures [38]. The BKMR model utilizes the Bayesian method. The Markov chain Monte Carlo (MCMC) algorithm screens the variables and constructs the Gaussian kernel function, which flexibly simulates the complex relationship between the response variable and multiple predictors and visualizes the potential interactions among these predictors [39]. The core formula of the BKMR model is as follows:

$$Y_{i}=h\left(Z_{i1},\ldots ,Z_{\mathrm{iM}}\right)+x_{i}\beta +\varepsilon_{i}$$

where Y_i_ represents health outcomes (categorical or continuous variables), Z is the exposure variable, h(…) denotes the constructed exposure–response function, $x_{i}$ and β represent the adjusted covariates and their corresponding coefficients, and ε_i_ is the residual term. For BKMR sampling, 5000 iterations were performed using the MCMC algorithm. Firstly, posterior inclusion probabilities (PIPs) of each metal under different lung function indicators were calculated using the BKMR model to determine the importance between metals and lung function indicators, with a lower value (close to 0) indicating a lower importance. Secondly, the overall correlation analysis between metal and lung function indicators was performed using the mixed exposure effect diagram. Thirdly, an influence diagram of single–metal exposure on lung function was illustrated to determine the correlation between single–metal exposure and lung function indexes by fixing other metals at the 25th, 50th, or 75th percentile and examining the link between individual metals and lung function indicator levels. Fourthly, when all other metals were fixed at the 50th percentile, the BKMR model was used to generate a univariate exposure–response curve to elucidate the potential non–linear relationship among the metals and lung function indicators. Finally, bivariate exposure–response curves of metal and lung function indicators were plotted using the BKMR model to explore the potential interactions among metals. The adjusted covariates in BKMR were the same as those in the multiple linear regression analysis.

The results of the regression analysis were expressed as coefficients and their corresponding 95% confidence intervals (CIs). The statistical significance level was set at 0.05 (two–tailed). All statistical analyses were conducted using R 4.2.1 (R Development Core Team, University of Auckland, New Zealand). BKMR and Qgcomp models were implemented with the R packages “bkmr” (version 0.2.2) and “qgcomp” (version 2.8.6), respectively.

## 3. Results

### 3.1. Population Characteristics

In total, 4382 participants who completed the examination were included. The general characteristics of all participants are listed in Table 1. Among all participants, there were marginally fewer males than females (49.1% vs. 50.9%). Furthermore, the serum cotinine level of the children and adolescents group was considerably lower than that of the adult and the elderly group. With the increase in age, the first and second baseline time and FET decreased. Similarly, we found statistical differences in terms of gender, race, the poverty income ratio, BMI, physical activity, and serum cotinine level. Further information is mentioned in Table 1. 

As shown in Table 2, the detection rates of urinary As and its metabolites (total As and DMA) and urinary metal metabolites (Ba, Ca, Co, Ce, Mo, Pb, Tl, W, U, and Hg) were higher than 80%. Furthermore, only AsB (57.89%) and Sb (70.63%) had detection rates of less than 80%. Following grouping according to age, the metals and their metabolites in the adult and elderly groups were identical to those in the total study population. However, the detection rate of Sb in the children and adolescents group was similarly higher than 80% (Tables S1–S3).

### 3.2. Urinary Metal Distribution, Correlation, and Selection

**Figure 2:**
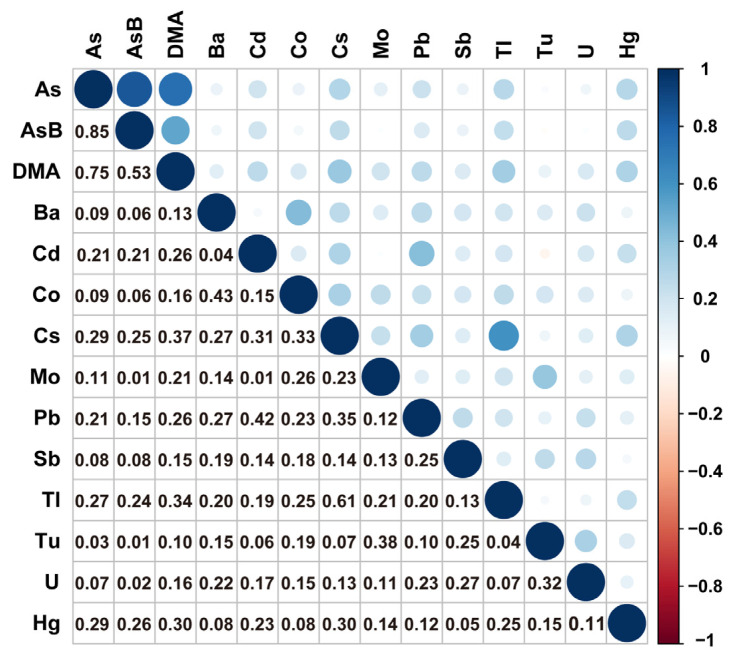
The correlations among the urinary concentrations in the 14 metals tested.Pearson correlation was performed to determine the correlations among the urinary concentrations in the 14 metals. The black numbers in the lower-left part indicate the correlation coefficients. The upper–right part indicates the heat map of the correlation coefficients between chemical concentrations. The white part represents no correlation (r = 0.00), blue represents a positive correlation, and red represents a negative correlation. The darker the color, the greater the correlation coefficient.

The Pearson correlation results showed a correlation among the metals of 4382 participants (*p* < 0.05). We found a strong correlation between Ce and Tl (r = 0.61), As and DMA (r = 0.75), and As and AsB (r = 0.85) (Figure 2).

**Figure 3:**
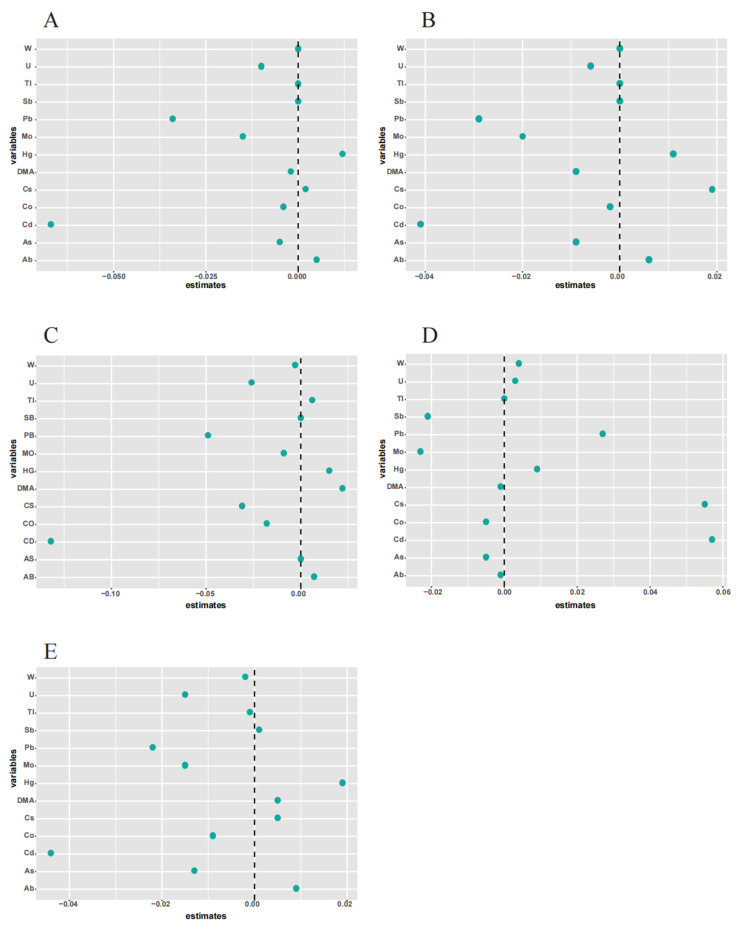
Estimated coefficients of the association between metals and pulmonary function by elastic net regression models. The dotted line represents 0, and each circle represents each metal substance. The farther the circle is from the dotted line, the greater the correlation between metal and lung function.The model was adjusted for gender, race/ethnicity, BMI, family income poverty ratio, physical activity, and serum cotinine levels. The correlation between a metal and lung function becomes stronger as the point deviates from 0. (**A**) FEV1, (**B**) FVC, (**C**) FEF25–75%, (**D**) FET, and (**E**) PEF.

Subsequently, we used an ENET model to identify elements in the metal mixtures in the environment that play an important role in lung function (Figure 3). Furthermore, the results showed that As and its metabolites, namely, Pb, Mo, Cd, U, Hg, and Cs, were correlated with all lung functions (*p* < 0.05). Cd was significantly negatively correlated with FEV1, FVC, FEF25–75%, and PEF and positively correlated with FET. Hg was significantly positively correlated with all lung functions (*p* < 0.05). Based on the results of ENET, we selected urinary metals and their metabolites associated with each of the five lung function indices and added them into a subsequent mixed exposure model to determine possible correlations with lung functions.

### 3.3. Association between Metal Exposure and Lung Functions

The outcomes of the generalized linear model on the correlation between metals and the outcomes of lung functions are shown in Table S4. Altogether, the model adjusted for all covariates showed that Pb and Cd were negatively correlated with lung functions, whereas Ba was positively correlated with lung functions, regardless of age (*p* < 0.05). In the children and adolescent group, Pb was negatively correlated with the rest of the lung function indicators except for FET. In the adult group, Cd was positively correlated with FET and negatively correlated with the other indicators. However, in the older age group, Cd showed a negative correlation with lung function except for FET, whereas Ba showed a positive correlation (*p* < 0.05). The uncorrected model is shown in Table S5.

**Figure 4:**
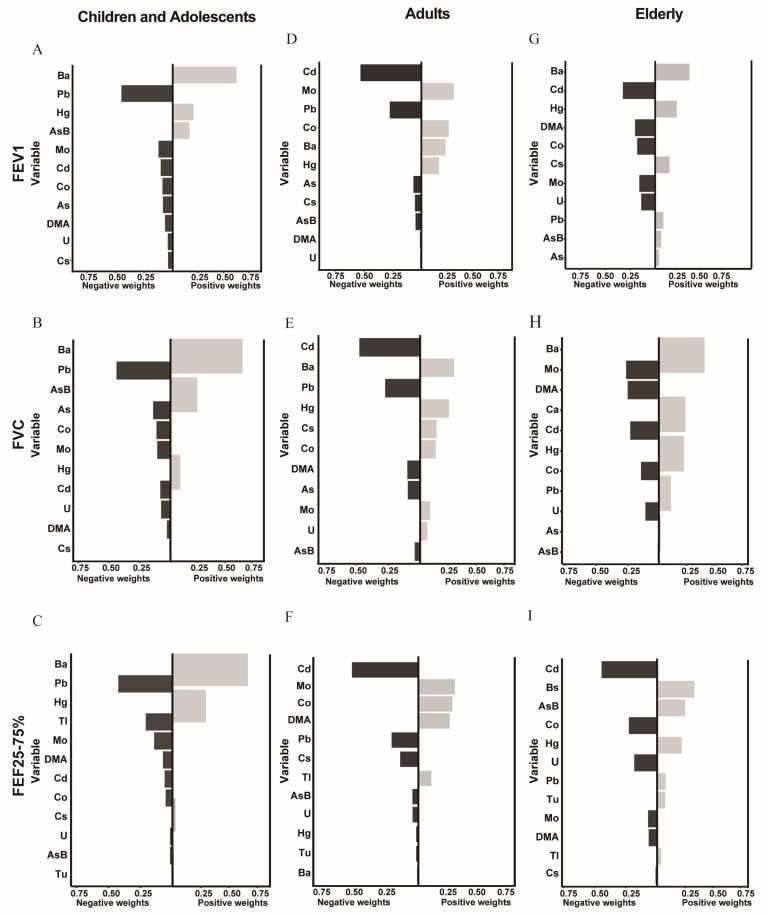
The correlation between the Qgcomp index and pulmonary function. The model was adjusted for gender, race/ethnicity, BMI, family income poverty ratio, physical activity, and serum cotinine levels. Figure (**A**–**C**) show the children and adolescents group; figure (**D**–**F**) show the adult group; and figure (**G**–**I**) show the elderly group.

Qgcomp regression analysis was performed to determine the correlation between mixed metal exposure and the lung function indicators. Furthermore, the results of this model are consistent with our earlier findings (Figure 4 and Figure S1). In the group of children and adolescents, the Qgcomp results showed a potential negative correlation between the concentration of mixed heavy metal exposures and the lung function indices, namely, FEV1, FVC, FET, PEF, and FEF25–75% (*p* < 0.05). In the adult group, all lung function indices except FET showed a negative correlation (*p* < 0.05) with mixed heavy metal concentrations, and the same results were observed in the elderly group.

For the FEV1 index, Cd and DMA showed a stable negative correlation in the three age groups. The greatest weight was given to Pb in the children and adolescents group, with a decrease of 0.45 units in FEV1 for a one–unit increase in Pb. Cd is the most crucial heavy metal in the adult and older age groups, with a decrease of 0.53 and 0.32 units of FEV1 for a one–unit increase in Cd, respectively. On the other hand, Ba showed a stable positive correlation in all age groups (*p* < 0.05). Among the metals negatively correlated with FVC, Pb (0.26), Cd (0.48), and Mo (0.44) exhibited the highest weights. Furthermore, FEF25–75% was negatively correlated with heavy metals, which was the same as that for FEV1. In the case of FET, Cs was positively correlated, regardless of age. Ba showed a stable positive correlation with PET in all age groups. As showed a stable negative correlation with PET.

### 3.4. Association among Urinary Metals and Lung Functions Using the BMKR Model

**Figure 5:**
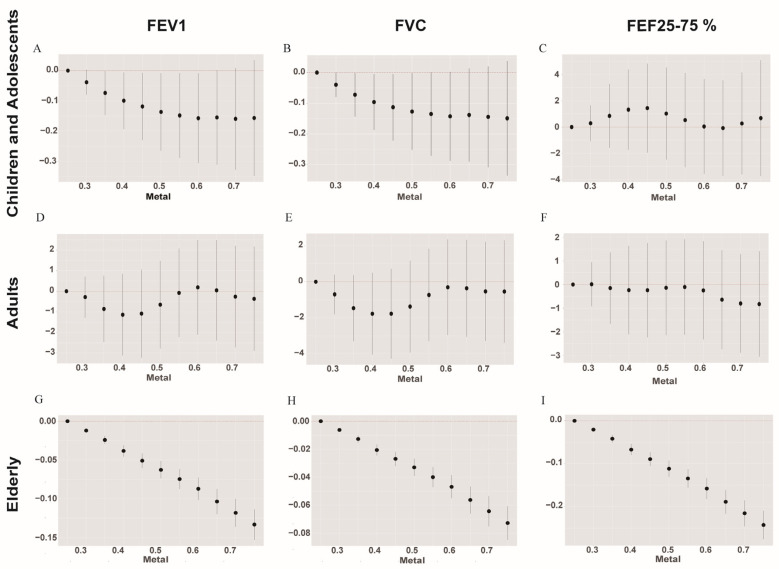
Association between metal mixtures and lung functions, as estimated by the BKMR model. The overall effect of metal and lung function (95%CI). Figure (**A**–**C**) show the children and adolescents group; figure (**D**–**F**) show the adult group; and figure (**G**–**I**) show the elderly group. The model adjusted urinary creatinine, gender, race, family income poverty ratio, BMI, serum cotinine, and physical activity.

The BKMR model was used to determine the association between metal mixtures and lung functions. Initially, the results of the mixed exposure effect plot showed a significant negative correlation between the lung function indices FEV1 and FVC when adolescent children and elderly people were exposed to metal mixtures relative to the adult group. Additionally, we found that metal mixture exposure was positively correlated with FET regardless of the age group (*p* < 0.05), indicating that baseline FET is associated with the amount of metal exposure (Figure 5 and Figure S2). We found that for young children and adults, the older age group was more sensitive to metal exposure. Furthermore, all five lung function indices significantly decreased after metal mixture exposure. 

**Figure 6:**
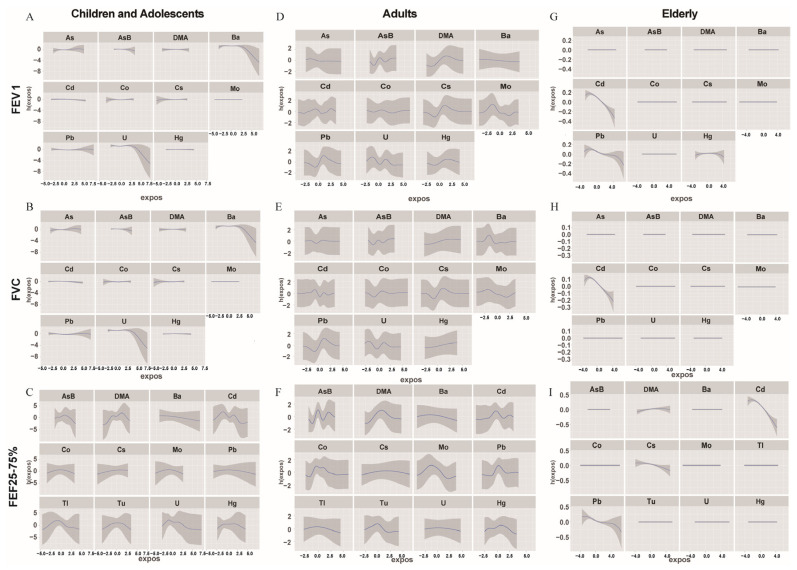
Association among metal mixtures and lung functions, as estimated by the BKMR model. The univariate exposure–response function was determined by BKMR between each metal and lung function while the concentrations of other metals were fixed at median percentile values. Figure (**A**–**C**) show the children and adolescents group; figure (**D**–**F**) show the adult group; and figure (**G**–**I**) show the elderly group. The model adjusted urinary creatinine, gender, race, family income poverty ratio, BMI, serum cotinine levels, and physical activity.

We further investigated the interactions among these metals. Fixing the remaining metals at the median level, we determined the exposure–response relationship between a particular metal and different lung functions by fixing another metal at the 25th, 50th, and 75th percentiles, respectively. The effects of individual metal variables on lung functions were determined by univariate exposure–response function curves and effect plots of single–metal exposure on lung functions, and the relative importance of each metal variable was reflected by PIP (Figure 6 and Figure S3). Some differences in the three subgroups were observed. Notably, Pb (PIP = 0.948), U (PIP = 0.102), and Ba (PIP = 0.106) were the key metals causing changes in the lung functions of the children and adolescent groups. Furthermore, Pb (PIP = 1.000), Cd (PIP = 0.864), and Hg (PIP = 0.110) were the key metals causing changes in the lung functions of the adult group. Lastly, Pb (PIP = 1.000) and Cd (PIP = 1.000) were the key metals causing changes in the lung functions of the older group. When other metals were fixed at the 25th, 50th, and 75th percentiles, Ba had a negative effect on the lung functions of the children and adolescents group and Ca had a negative effect on the lung function of the elderly group. However, in the adult group, no metal played a predominant role in lung functions, indicating that the metals interacted with each other. Furthermore, as shown in Figure 5 and Figure 6, we found that Pb, As, and DMA played an important role in affecting lung functions because they were stable in different age groups for individual and mixed exposures (*p* < 0.05) (Figures S1–S3).

Lastly, all the metals were at their 50th percentile, and the interactions between the metals were analyzed by the bivariate exposure–response function of the BKMR model. The slopes of the bivariate metal response functions for one metal metabolite were similar at different quartiles for the other metal metabolite, which suggested no interaction. The results suggest that certain interactions are present among the metals at any stage (Figures S4–S7).

## 4. Discussion

Due to the potential adverse health effects associated with exposure to environmentally hazardous metals, countries consider it a major public health concern that demands immediate attention. The inhalation of metal-containing pollutants by humans can contribute to acute chemical pneumonia, pulmonary edema, or acute trachea and bronchitis [40]. Therefore, the relationship between metal exposure and lung function has garnered increasing global attention. In this study, four statistical methods were used to assess the relationship between exposure to mixed metals at different life stages and five lung function indicators. Altogether, we found that, in comparison with children, adolescents, and the elderly, adults exposed to metal mixtures have more metals affecting lung functions. Furthermore, Pb plays an important role in the effects of metal exposure on lung function across all ages.

Because metals are usually interrelated in the environment, they can exhibit mutual promotion or inhibition, suggesting synergistic or antagonistic effects on human health [41,42,43]. In this study, several models were used to determine the effects of mixed metal exposure on lung functions. Different models have distinct advantages and disadvantages, making the results more compatible and accurate. The GLM model can reveal comparatively direct and easily explainable results [44,45]. Qgcomp can determine the weight of each component in mixed exposure, whereas BKMR can determine the risk of the entire mixture and the nonlinear relationship and interaction among the constituent mixtures [46,47,48].

As is a ubiquitous metalloid [49]. Even though As is not more toxic than the other metals listed in the Toxic Substances and Disease Registry list, it ranks higher than lead, mercury, and cadmium based on its exposure frequency, toxicity, and human exposure potential [50]. As is known to cause lung cancer. As exposure plays an important role in diseases such as pulmonary fibrosis, lung injury, and lung inflammation. As exposure activates inflammatory markers [51]. In humans, As exposure is associated with increased levels of matrix metalloproteinase-9, a biomarker of lung inflammation [52]. In the present study, we found correlations between arsenic and its metabolite (DMA) and indicators of FEF25–75%, FET, and PEF in the different age groups of the participants. Notably, As is one of the few metals metabolized by the human body [53]. As compounds ingested by humans and rodents are detoxified and excreted after methylation, resulting in the presence of DMA [49,54,55], which is a product of arsenic methylation. DMA is larger and more persistent than As in the body. Therefore, DMA may be more sensitive to As exposure in vivo than total arsenic levels, and an increase in total As levels in the human body indicates recent excessive exposure to As [56].

We found that both Ba and Pb notably affected the children and adolescents group when exposed to metal mixtures. Pb is generally inhaled through the respiratory tract, but children are more exposed to Pb through oral chewing of Pb–containing items compared with individuals in other age groups [57,58]. A cross-sectional study indicated that urinary Pb is negatively correlated with FVC in children aged 6–17 who participated in NHANES [57]. The results of a small cohort study of 107 primary school children in Mongolia revealed that airborne Pb exposure is associated with reduced PEF [59]. Although there is a paucity of in vivo experiments on the effects of Pb on lung function, we found that both Pb and the cortisol damaged by Pb can independently improve the pro–inflammatory immune environment or oxidative state affecting the programming of lung development [58,60]. Even though there are few biological studies on Ba, epidemiological investigations suggest that Ba contributes to decreased lung functions [61]. Furthermore, Ba titanate nanoparticles can induce cytotoxicity in human lung cancer cells via oxidative stress [62].

In vivo experiments [63] have shown that chronic exposure to Cd in mice can contribute to COPD, and a decrease in characteristic lung functions can be observed. We found that Cd and Pb in metal mixtures were associated with all five lung function measurements in the adult group. Cd is a pulmonary toxin that causes respiratory diseases [64]. A previous study showed that the Cd-metalloproteome may contain 18.4% of the complete human proteome [65]. Furthermore, another study showed that Cd-metalloprotein is associated with lung adenocarcinoma [66]. The correlation between Cd levels in male blood and lung functions was confirmed by epidemiological and in vivo, respectively [67,68]. Cd can cause an anti–apoptotic survival response via many signaling pathways, contributing to cell death [69], repair and survival, or malignant transformation. Cd exposure can cause interstitial pulmonary fibrosis or emphysema, which is associated with the synthesis of connective tissue proteins [70]. Although Pb is associated with lung function in adults, it is more significant in children and adolescents who are more susceptible and more prone to environmental pollutants due to their growth and developmental characteristics, behavioral patterns, and lower awareness of the risks [71].

Cd, Pb, and Ba emerge as significant components affecting lung functions in response to metal mixtures, particularly in older age groups compared with children and adult groups. In contrast to other populations, Cs sensitivity is more pronounced in older age groups. Although Cs and lung-related studies are limited, some research has linked soil 137Cs to pediatric obstructive and restrictive lung function impairment [72]. The results of the present study are consistent with those of previous studies, which showed that children and older adults are more sensitive to metal exposure than adults. However, it is noteworthy that the metal species associated with lung functions were much higher in adults than in children and older adults for mixed metal exposures. Metals are fixed in the environment, but environmental metal distribution can be changed by anthropogenic activities, making adults, especially those in industrial occupations, more prone to metal exposure compared with children and the elderly. The interrelation among metals in the environment suggests potential synergistic or antagonistic effects on human health [41,42,43], posing greater health risks in mixed exposures. Children and the elderly benefit from protective policies against toxic substances, whereas less attention is paid to normal adults, making them the group with the highest exposure risk to toxic metal species [73].

Our study presents several advantages. Firstly, in contrast to previous investigations that focused solely on the correlation between individual metal exposure and lung function, we used multiple models to determine the relationship between metal exposure and lung function across different segments of the U.S. population. This approach aimed to improve the understanding of the effect of metals, whether in isolation or combination, on lung functions, as well as the specific role played by each metal in mixed exposure. Secondly, we used data from the U.S. Health and Nutrition Survey, providing a substantial and representative sample size. Thirdly, our study determined the effects of metal exposure on lung function within distinct stages of a unified sample, highlighting innovative approaches. However, our study has some limitations that should be addressed. Firstly, the cross–sectional design of the included data makes it difficult to establish a causal relationship between urinary metal levels and lung functions. Secondly, due to ethical issues, we were unable to obtain baseline metal levels in the population for comparison. Thirdly, the calculation of values below the LOD by dividing the square root of LOD by 2 may introduce bias [74]. Additionally, there is inevitably selection bias in the participant selection process, potentially affecting the results. Lastly, although our study involved many covariates, we did not conduct an in–depth exploration of other covariates. It is important to conduct an in–depth study of other covariates in possible follow-up studies to provide further insights for related research.

## 5. Conclusions

In this study, diverse statistical models were used to investigate the effects of metal exposure on lung functions across different age groups. The findings suggest that in children and adolescents, the primary contributors to the effects of metal mixture exposure on lung function were Pb and Ba. Conversely, for adults and the elderly, Cd played a major role. Additionally, the metabolites of As also influenced lung functions. Although children and the elderly exhibited the highest sensitivity to metals, adults had greater overall exposure. It is crucial to acknowledge the health damage resulting from metal exposure in adults, which also warrants attention.

## Figures and Tables

**Table 1:** Demographic characteristics of the study population, NHANES 2007–2012 (*n* = 4382).

	All Participants	Children and Adolescents (6–18)	Adults (19–59)	Elderly (≥60)
Gender n (%)				
Male	2217 (49.1)	245 (48.5)	1454 (49.9)	518 (45.9)
Female	2165 (50.9)	235 (51.5)	1431 (50.1)	499 (54.1)
Race n (%)				
Mexican American	750 (8.4)	132 (14.0)	497 (9.0)	121 (3.6)
Other Hispanic	461 (5.5)	47 (4.8)	311 (6.1)	103 (3.1)
Non-Hispanic White	1888 (68.9)	164 (61.4)	1225 (66.9)	499 (80.3)
Non-Hispanic Black	909 (10.7)	110 (14.0)	566 (10.9)	233 (8.4)
Other race—including multi-racial	374 (6.5)	27 (5.8)	286 (7.1)	61 (4.6)
Family income poverty ratio n (%)				
<1.3	1382 (20.3)	187 (26.8)	919 (20.8)	276 (15.8)
1.3–3.5	1652 (35.9)	182 (36.6)	1048 (34.7)	422 (40.2)
>3.5	1348 (43.8)	111 (36.6)	918 (44.5)	319 (44.0)
Recreational activities n (%)				
Yes	2397 (61.0)	406 (88.1)	1567 (61.2)	424 (48.6)
No	1985 (39.0)	74 (11.9)	1318 (38.8)	593 (51.4)
Body mass index (BMI) n (%)				
<18.5 kg/m^2^	137 (2.6)	74 (15.9)	55 (1.7)	8 (0.6)
18.5 < 25 kg/m^2^	1373 (33.0)	256 (57.6)	896 (33.1)	221 (22.7)
25 < 30 kg/m^2^	1411 (32.7)	84 (14.8)	939 (33.1)	388 (38.5)
≥30 kg/m^2^	1461 (31.7)	66 (11.7)	995 (32.1)	400 (38.2)
Pulmonary function Mean (SD)				
FEV1 (mL)	8.29 (0.27)	8.26 (0.22)	8.34 (0.25)	8.09 (0.29)
FVC (mL)	8.05 (0.30)	8.11 (0.23)	8.11 (0.26)	7.77 (0.31)
FEF25–75% (mL/s)	8.98 (0.29)	8.90 (0.22)	9.03 (0.26)	8.81 (0.33)
PEF (mL/s)	7.93 (0.52)	8.19 (0.32)	8.03 (0.43)	7.41 (0.58)
FET (s)	2.30 (0.37)	1.92 (0.39)	2.29 (0.33)	2.50 (0.35)
NHANES cycles n (%)				
2007–2008	1446 (32.9)	206 (47.9)	885 (31.7)	355 (31.5)
2009–2010	1751 (35.6)	274 (52.1)	1104 (34.3)	373 (34.2)
2011–2012	1185 (31.5)	0 (0.0)	896 (34.0)	289 (34.3)

**Table 2:** Urinary metal distribution in the study population, NHANES 2007–2012 (*n* = 4382). Adjusting for ln-transformed urinary creatinine.

Metal Metabolites	Detection Rate N (%)	Mean	LOD	Percentiles				
				P5	P25	P50	P75	P95
Urinary total arsenic	4326 (98.72)	16.90	0.26	2.38	4.28	6.97	14.84	53.30
Urinary arsenobetaine	2537 (57.89)	8.72	1.19	0.16	0.48	1.49	5.49	35.87
Urinary dimethylarsonic acid	3577 (81.63)	4.95	1.91	1.40	2.34	3.51	5.73	12.39
Urinary barium	4361 (99.52)	2.29	0.06	0.36	0.85	1.49	2.62	6.14
Urinary cadmium	4020 (91.74)	0.30	0.04	0.05	0.11	0.19	0.36	0.86
Urinary cobalt	4358 (99.45)	0.48	0.02	0.14	0.23	0.34	0.51	1.13
Urinary cesium	4382 (100.0)	4.96	0.09	1.98	3.14	4.27	5.98	9.90
Urinary molybdenum	4380 (99.95)	50.04	0.08	14.94	27.75	40.56	60.42	114.25
Urinary lead	4242 (96.81)	0.59	0.03	0.15	0.28	0.43	0.68	1.45
Urinary antimony	3095 (70.63)	0.08	0.02	0.02	0.04	0.06	0.08	0.19
Urinary thallium	4356 (99.41)	0.18	0.02	0.07	0.11	0.15	0.21	0.37
Urinary tungsten	3846 (87.77)	0.12	0.02	0.02	0.05	0.08	0.14	0.37
Urinary uranium	3787 (86.42)	0.01	0.01	0.00	0.00	0.01	0.01	0.03
Urinary mercury	4382 (100.0)	0.69	0.13	0.10	0.21	0.43	0.82	2.12
Serum cotinine	4382 (100.0)	51.74	0.01	0.01	0.02	0.05	4.07	328.70

## Data Availability

The data was retrieved from publicly available resources and can be accessed from National Center for Health Statistics of Center for Disease Control and Prevention through https://www.cdc.gov/nchs/nhanes/index.htm.

## Supplementary Materials

The following supporting information can be downloaded at: https://www.mdpi.com/article/10.3390/metabo14030139/s1, **Figure S1:** The correlation between the Qgcomp index and pulmonary function. The model was adjusted for gender, race/ethnicity, BMI, family income poverty ratio, physical activity, and serum cotinine levels. Figure (**A**,**B**) show the children and adolescents group; figure (**C**,**D**) show the adult group; and figure (**E**,**F**) show the elderly group; **Figure S2:** Association between metal mixtures and lung functions, as estimated by the BKMR model. The overall effect of metal and lung function (95%CI). Figure (**A**,**B**) show the children and adolescents group; figure (**C**,**D**) show the adult group; and figure (**E**,**F**) show the elderly group. The model adjusted urinary creatinine, gender, race, family income poverty ratio, BMI, serum cotinine, and physical activity; **Figure S3:** Association among metal mixtures and lung functions, as estimated by the BKMR model. The univariate exposure–response function was determined by BKMR between each metal and lung function while the concentrations of other metals were fixed at median percentile values; Figure (**A**,**B**) show the children and adolescents group; figure (**C**,**D**) show the adult group; and figure (**E**,**F**) show the elderly group. The model adjusted urinary creatinine, gender, race, family income poverty ratio, BMI, serum cotinine levels, and physical activity; **Figure S4:** Association between metal mixtures and lung function estimated by the BKMR model. The figure describe the estimated difference in lung function from 25th to 75th percentiles for each metal when the other metal is fixed at 25th percentile (red line), 50th percentile (green line), or 75th percentile (blue line). This point represents the estimated value, and the horizontal line represents the 95% confidence interval (CI). The model adjusted urinary creatinine, gender, race, PIR, BMI, serum cotinine and physical activity. Figure (**A**–**E**) show the children and adolescents group; figure (**F**–**J**) show the adult group; and figure (**K**–**O**) show the elderly group; **Figure S5:** Association between metal mixtures and lung function in children and adolescents group estimated by the BKMR model. The figure are bivariate exposure response functions of metal and lung function. When one metal is fixed at different (25, 55, 75) percentiles, while the other metal is fixed at the 50th percentile, the average difference between the other metal and the lung function as a bivariate exposure response function. The model adjusted urinary creatinine, gender, race, PIR, BMI, serum cotinine and physical activity; **Figure S6:** Association between metal mixtures and lung function in adults group estimated by the BKMR model. The figure are bivariate exposure response functions of metal and lung function. When one metal is fixed at different (25, 55, 75) percentiles, while the other metal is fixed at the 50th percentile, the average difference between the other metal and the lung function as a bivariate exposure response function. The model adjusted urinary creatinine, gender, race, PIR, BMI, serum cotinine and physical activity. **Figure S7:** Association between metal mixtures and lung function in elderly peoples group estimated by the BKMR model. The figure are bivariate exposure response functions of metal and lung function. When one metal is fixed at different (25, 55, 75) percentiles, while the other metal is fixed at the 50th percentile, the average difference between the other metal and the lung function as a bivariate exposure response function. The model adjusted urinary creatinine, gender, race, PIR, BMI, serum cotinine and physical activity. Table S1: Urinary Metal Distribution in Children and Adolescents of the study population, NHANES 2007–2012 (n = 4382); Table S2: Urinary Metal Distribution in Adults of the study population, NHANES 2007–2012 (n = 4382); Table S3: Urinary Metal Distribution in the elderly of the study population, NHANES 2007–2012 (n = 4382); Table S4: Relationship between metal and lung function of the study population, NHANES 2007–2012 (n = 4382); Notes: FEV1, forced expiratory volume in 1s; FVC, forced vital capacity; FEF25–75%, forced expiratory fow between 25 and 75% of FVC; PEF, peak expiratory fow rate. FET, forced expiratory time. Adjusted for age, sex, race/ethnicity, education level, marital status, ratio of family income to poverty, BMI, physical activity level, alcohol status and smoking status. ^a^ P < 0.05; Table S5: Relationship between metal and lung function of the study population, NHANES 2007–2012 (n = 4382); Notes: FEV1, forced expiratory volume in 1s; FVC, forced vital capacity; FEF25–75%, forced expiratory fow between 25 and 75% of FVC; PEF, peak expiratory fow rate. ^a^ P < 0.05.

## References

[1] Vareda J.P., Valente A.J.M., Durães L. (2019). Assessment of heavy metal pollution from anthropogenic activities and remediation strategies: A review. J. Environ. Manag..

[2] Chen L., Sun Q., Peng S., Tan T., Mei G., Chen H., Zhao Y., Yao P., Tang Y. (2022). Associations of blood and urinary heavy metals with rheumatoid arthritis risk among adults in NHANES 1999–2018. Chemosphere.

[3] Witkowska D., Słowik J., Chilicka K. (2021). Heavy Metals and Human Health: Possible Exposure Pathways and the Competition for Protein Binding Sites. Molecules.

[4] Danziger J., Dodge L.E., Hu H., Mukamal K.J. (2022). Susceptibility to Environmental Heavy Metal Toxicity among Americans with Kidney Disease. Kidney360.

[5] Kiani B., Amin F.H., Bagheri N., Bergquist R., Mohammadi A.A., Yousefi M., Faraji H., Roshandel G., Beirami S., Rahimzadeh H. (2021). Association between heavy metals and colon cancer: An ecological study based on geographical information systems in North-Eastern Iran. BMC Cancer.

[6] Nguyen H.D., Oh H., Hoang N.H.M., Jo W.H., Kim M.-S. (2021). Environmental science and pollution research role of heavy metal concentrations and vitamin intake from food in depression: A national cross-sectional study (2009–2017). Environ. Sci. Pollut. Res..

[7] Guo X., Li N., Wang H., Su W., Song Q., Liang Q., Liang M., Sun C., Li Y., Lowe S. (2022). Combined exposure to multiple metals on cardiovascular disease in NHANES under five statistical models. Environ. Res..

[8] Haddi A.A.A., Jaafar M.H., Ismail H. (2022). Association between lung function impairment with urinary heavy metals in a community in Klang Valley, Malaysia. PeerJ.

[9] Dwyer-Lindgren L., Bertozzi-Villa A., Stubbs R.W., Morozoff C., Shirude S., Naghavi M., Mokdad A.H., Murray C.J.L. (2017). Trends and Patterns of Differences in Chronic Respiratory Disease Mortality Among US Counties, 1980–2014. JAMA.

[10] Sobel M., Navas-Acien A., Powers M., Grau-Perez M., Goessler W., Best L.G., Umans J., Oelsner E.C., Podolanczuk A., Sanchez T.R. (2021). Environmental-level exposure to metals and metal-mixtures associated with spirometry-defined lung disease in American Indian adults: Evidence from the Strong Heart Study. Environ. Res..

[11] Assad N., Sood A., Campen M.J., Zychowski K.E. (2018). Metal-Induced Pulmonary Fibrosis. Curr. Environ. Health Rep..

[12] Wu L., Cui F., Ma J., Huang Z., Zhang S., Xiao Z., Li J., Ding X., Niu P. (2022). Associations of multiple metals with lung function in welders by four statistical models. Chemosphere.

[13] Oh J., Shin S.H., Choi R., Kim S., Park H.-D., Kim S.-Y., Han S.A., Koh W.-J., Lee S.-Y. (2019). Assessment of 7 trace elements in serum of patients with nontuberculous mycobacterial lung disease. J. Trace Elements Med. Biol..

[14] Wu W., Bromberg P.A., Samet J.M. (2013). Zinc ions as effectors of environmental oxidative lung injury. Free. Radic. Biol. Med..

[15] Chang Z., Qiu J., Wang K., Liu X., Fan L., Liu X., Zhao Y., Zhang Y. (2023). The relationship between co-exposure to multiple heavy metals and liver damage. J. Trace Elements Med. Biol..

[16] Andjelkovic M., Djordjevic A.B., Antonijevic E., Antonijevic B., Stanic M., Kotur-Stevuljevic J., Spasojevic-Kalimanovska V., Jovanovic M., Boricic N., Wallace D. (2019). Toxic Effect of Acute Cadmium and Lead Exposure in Rat Blood, Liver, and Kidney. Int. J. Environ. Res. Public Health.

[17] Wang X., Mukherjee B., Park S.K. (2018). Associations of cumulative exposure to heavy metal mixtures with obesity and its comorbidities among U.S. adults in NHANES 2003–2014. Environ. Int..

[18] Di Cicco M., Kantar A., Masini B., Nuzzi G., Ragazzo V., Peroni D. (2020). Structural and functional development in airways throughout childhood: Children are not small adults. Pediatr. Pulmonol..

[19] Smith L.S., Zimmerman J.J., Martin T.R. (2013). Mechanisms of acute respiratory distress syndrome in children and adults: A review and suggestions for future research. Pediatr. Crit. Care Med..

[20] Rissler J., Gudmundsson A., Nicklasson H., Swietlicki E., Wollmer P., Löndah J. (2017). Deposition efficiency of inhaled particles (15–5000 nm) related to breathing pattern and lung function: An experimental study in healthy children and adults. Part. Fibre. Toxicol..

[21] Liang T.I., Lee E.Y. (2020). Interstitial Lung Diseases in Children, Adolescents, and Young Adults: Different from Infants and Older Adults. Radiol. Clin. N. Am..

[22] Zhang Z., Guo L., Huang L., Zhang C., Luo R., Zeng L., Liang H., Li Q., Lu X., Wang X. (2021). Distinct Disease Severity Between Children and Older Adults with Coronavirus Disease 2019 (COVID-19): Impacts of ACE2 Expression, Distribution, and Lung Progenitor Cells. Clin. Infect. Dis..

[23] Sharma G., Goodwin J. (2006). Effect of aging on respiratory system physiology and immunology. Clin. Interv. Aging.

[24] Tian X., Xue B., Wang B., Lei R., Shan X., Niu J., Luo B. (2022). Physical activity reduces the role of blood cadmium on depression: A cross-sectional analysis with NHANES data. Environ. Pollut..

[25] Yang X., Xue Q., Wen Y., Huang Y., Wang Y., Mahai G., Yan T., Liu Y., Rong T., Wang Y. (2022). Environmental polycyclic aromatic hydrocarbon exposure in relation to metabolic syndrome in US adults. Sci. Total Environ..

[26] Hu P., Su W., Vinturache A., Gu H., Cai C., Lu M., Ding G. (2020). Urinary 3-phenoxybenzoic acid (3-PBA) concentration and pulmonary function in children: A National Health and Nutrition Examination Survey (NHANES) 2007–2012 analysis. Environ. Pollut..

[27] Shi Y., Wang H., Zhu Z., Ye Q., Lin F., Cai G. (2023). Association between exposure to phenols and parabens and cognitive function in older adults in the United States: A cross-sectional study. Sci. Total Environ..

[28] Che Z., Jia H., Chen R., Pan K., Fan Z., Su C., Wu Z., Zhang T. (2023). Associations between exposure to brominated flame retardants and metabolic syndrome and its components in U.S. adults. Sci. Total Environ..

[29] Li K., Yin R., Wang Y., Zhao D. (2021). Associations between exposure to polycyclic aromatic hydrocarbons and metabolic syndrome in U.S. adolescents: Cross-sectional results from the National Health and Nutrition Examination Survey (2003–2016) data. Environ. Res..

[30] Peng K., Li Z., Gao T.-R., Lv J., Wang W.-J., Zhan P., Yao W.-C., Zhao H., Wang H., Xu D.-X. (2023). Polycyclic aromatic hydrocarbon exposure burden: Individual and mixture analyses of associations with chronic obstructive pulmonary disease risk. Environ. Res..

[31] Guo X., Wu B., Hu W., Wang X., Su W., Meng J., Lowe S., Zhao D., Huang C., Liang M. (2023). Associations of perchlorate, nitrate, and thiocyanate with metabolic syndrome and its components among US adults: A cross-sectional study from NHANES. Sci. Total Environ..

[32] Kang H., Lee J.P., Choi K. (2021). Exposure to phthalates and environmental phenols in association with chronic kidney disease (CKD) among the general US population participating in multi-cycle NHANES (2005–2016). Sci. Total Environ..

[33] Kim S.S., Meeker J.D., Carroll R., Zhao S., Mourgas M.J., Richards M.J., Aung M., Cantonwine D.E., McElrath T.F., Ferguson K.K. (2018). Urinary trace metals individually and in mixtures in association with preterm birth. Environ. Int..

[34] Teppola P., Taavitsainen V.-M. (2013). Parsimonious and robust multivariate calibration with rational function Least Absolute Shrinkage and Selection Operator and rational function Elastic Net. Anal. Chim. Acta.

[35] Vuong A.M., Xie C., Jandarov R., Dietrich K.N., Zhang H., Sjödin A., Calafat A.M., Lanphear B.P., McCandless L., Braun J.M. (2020). Prenatal exposure to a mixture of persistent organic pollutants (POPs) and child reading skills at school age. Int. J. Hyg. Environ. Health.

[36] Keil A.P., Buckley J.P., O’brien K.M., Ferguson K.K., Zhao S., White A.J. (2020). A Quantile-Based g-Computation Approach to Addressing the Effects of Exposure Mixtures. Environ. Health Perspect..

[37] Lee K.-S., Kim K.-N., Ahn Y.D., Choi Y.-J., Cho J., Jang Y., Lim Y.-H., Kim J.I., Shin C.H., Lee Y.A. (2021). Prenatal and postnatal exposures to four metals mixture and IQ in 6-year-old children: A prospective cohort study in South Korea. Environ. Int..

[38] Bobb J.F., Henn B.C., Valeri L., Coull B.A. (2018). Statistical software for analyzing the health effects of multiple concurrent exposures via Bayesian kernel machine regression. Environ. Health.

[39] Kim S.S., Meeker J.D., Keil A.P., Aung M.T., Bommarito P.A., Cantonwine D.E., McElrath T.F., Ferguson K.K. (2019). Exposure to 17 trace metals in pregnancy and associations with urinary oxidative stress biomarkers. Environ. Res..

[40] Nemery B. (1990). Metal toxicity and the respiratory tract. Eur. Respir. J..

[41] Huang C., Gao E., Xiao F., Wu Q., Liu W., Luo Y., Ren X., Chen X., He K., Huang H. (2023). The relative and interactive effects of urinary multiple metals exposure on hyperuricemia among urban elderly in China. Front. Public Health.

[42] Braun J.M., Gennings C., Hauser R., Webster T.F. (2016). What Can Epidemiological Studies Tell Us about the Impact of Chemical Mixtures on Human Health?. Environ. Health Perspect..

[43] Ren J., Jin H., Zhang C., Liu S., Han Y., Xi J., Cao J. (2023). Mixed exposure effect of seminal metals on semen quality, mediation of total antioxidant capacity, and moderation of GSTM1/GSTT1 gene deletion in Chinese reproductive-aged men. Environ. Res..

[44] Chang J.W. (2019). Risk factor analysis for the development and progression of retinopathy of prematurity. PLoS ONE.

[45] Su L., Ho H., Stock C.T., Quadri S.M., Williamson C., Servais E.L. (2021). Surgeon Experience Is Associated with Prolonged Air Leak After Robotic-assisted Pulmonary Lobectomy. Ann. Thorac. Surg..

[46] Li Z., Xu Y., Huang Z., Wei Y., Hou J., Long T., Wang F., Hu H., Duan Y., Guo H. (2019). Association between exposure to arsenic, nickel, cadmium, selenium, and zinc and fasting blood glucose levels. Environ. Pollut..

[47] Luo J., Hendryx M. (2020). Metal mixtures and kidney function: An application of machine learning to NHANES data. Environ. Res..

[48] Xu C., Liang J., Xu S., Liu Q., Xu J., Gu A. (2020). Increased serum levels of aldehydes are associated with cardiovascular disease and cardiovascular risk factors in adults. J. Hazard. Mater..

[49] Saintilnord W.N., Fondufe-Mittendorf Y. (2021). Arsenic-induced epigenetic changes in cancer development. Semin. Cancer Biol..

[50] Garbinski L.D., Rosen B.P., Chen J. (2019). Pathways of arsenic uptake and efflux. Environ. Int..

[51] Selman M., Pardo A. (2012). Alveolar epithelial cell disintegrity and subsequent activation: A key process in pulmonary fibrosis. Am. J. Respir. Crit. Care Med..

[52] Atkinson J.J., Senior R.M. (2003). Matrix Metalloproteinase-9 in Lung Remodeling. Am. J. Respir. Cell Mol. Biol..

[53] Moe B., Peng H., Lu X., Chen B., Chen L.W., Gabos S., Li X.-F., Le X.C. (2016). Comparative cytotoxicity of fourteen trivalent and pentavalent arsenic species determined using real-time cell sensing. J. Environ. Sci..

[54] R Roy N.K., Murphy A., Costa M. (2020). Arsenic Methyltransferase and Methylation of Inorganic Arsenic. Biomolecules.

[55] El-Ghiaty M.A., El-Kadi A.O. (2023). The Duality of Arsenic Metabolism: Impact on Human Health. Annu. Rev. Pharmacol. Toxicol..

[56] Cohen S.M., Arnold L.L., Beck B.D., Lewis A.S., Eldan M. (2013). Evaluation of the carcinogenicity of inorganic arsenic. Crit. Rev. Toxicol..

[57] Metryka E., Chibowska K., Gutowska I., Falkowska A., Kupnicka P., Barczak K., Chlubek D., Baranowska-Bosiacka I. (2018). Lead (Pb) Exposure Enhances Expression of Factors Associated with Inflammation. Int. J. Mol. Sci..

[58] Liu X., Zhang H., Zhang Y., Wang J., Tan H., Piao J., Yang L., Yang X. (2022). The Time Trend of Blood Lead and Cadmium Levels in Rural Chinese Children: China Nutrition and Health Survey 2002 and 2012. Biol. Trace Element Res..

[59] Madaniyazi L., Guo Y., Ye X., Kim D., Zhang Y., Pan X. (2013). Effects of Airborne Metals on Lung Function in Inner Mongolian Schoolchildren. J. Occup. Environ. Med..

[60] Rosa M.J., Tamayo-Ortiz M., Garcia A.M., Rivera N.Y.R., Bush D., Lee A.G., Solano-González M., Amarasiriwardena C., Téllez-Rojo M.M., Wright R.O. (2021). Prenatal lead exposure and childhood lung function: Influence of maternal cortisol and child sex. Environ. Res..

[61] Xiao L.L., Zhou Y., Cui X.Q., Huang X.J., Yuan J., Chen W.H. (2016). Association of urinary metals and lung function in general Chinese population of Wuhan. Zhonghua Yu Fang Yi Xue Za Zhi.

[62] Ahamed M., Akhtar M.J., Khan M.M., Alhadlaq H.A., Alshamsan A. (2020). Barium Titanate (BaTiO_3_) Nanoparticles Exert Cytotoxicity through Oxidative Stress in Human Lung Carcinoma (A549) Cells. Nanomaterials.

[63] Wang W.-J., Peng K., Lu X., Zhu Y.-Y., Li Z., Qian Q.-H., Yao Y.-X., Fu L., Wang Y., Huang Y.-C. (2023). Long-term cadmium exposure induces chronic obstructive pulmonary disease-like lung lesions in a mouse model. Sci. Total Environ..

[64] Knoell D.L., Wyatt T.A. (2021). The adverse impact of cadmium on immune function and lung host defense. Semin. Cell Dev. Biol..

[65] Chasapis C.T. (2018). Shared gene-network signatures between the human heavy metal proteome and neurological disorders and cancer types. Metallomics.

[66] Peana M., Pelucelli A., Chasapis C.T., Perlepes S.P., Bekiari V., Medici S., Zoroddu M.A. (2022). Biological Effects of Human Exposure to Environmental Cadmium. Biomolecules.

[67] Larson-Casey J.L., Liu S., Pyles J.M., Lapi S.E., Saleem K., Antony V.B., Gonzalez M.L., Crossman D.K., Carter A.B. (2023). Impaired PPARγ activation by cadmium exacerbates infection-induced lung injury. J. Clin. Investig..

[68] Oh C.-M., Oh I.-H., Lee J.-K., Park Y.H., Choe B.-K., Yoon T.-Y., Choi J.-M. (2014). Blood cadmium levels are associated with a decline in lung function in males. Environ. Res..

[69] Hu X., Fernandes J., Jones D.P., Go Y.-M. (2017). Cadmium stimulates myofibroblast differentiation and mouse lung fibrosis. Toxicology.

[70] Niewoehner D.E., Hoidal J.R. (1982). Lung fibrosis and emphysema: Divergent responses to a common injury?. Science.

[71] Zeng X., Huo X., Xu X., Liu D., Wu W. (2020). E-waste lead exposure and children’s health in China. Sci. Total Environ..

[72] Svendsen E.R., Kolpakov I.E., Karmaus W.J.J., Mohr L.C., Vdovenko V.Y., McMahon D.M., Jelin B.A., Stepanova Y.I. (2015). Reduced Lung Function in Children Associated with Cesium 137 Body Burden. Ann. Am. Thorac. Soc..

[73] Sierra-Vargas M.P., Montero-Vargas J.M., Debray-García Y., Vizuet-De-Rueda J.C., Loaeza-Román A., Terán L.M. (2023). Oxidative Stress and Air Pollution: Its Impact on Chronic Respiratory Diseases. Int. J. Mol. Sci..

[74] Nie L., Chu H., Liu C., Cole S.R., Vexler A., Schisterman E.F. (2010). Linear Regression with an Independent Variable Subject to a Detection Limit. Epidemiology.

