# Supplementary material for: The Correlation between Metal Mixed Exposure and Lung Function in Different Ages of the Population
Source: Metabolites. 2024 Feb 26;14(3):139. doi: 10.3390/metabo14030139 (PMC10972184; doi:10.3390/metabo14030139)
Supplement: Supplementary file 1 [file metabolites-14-00139-s001.zip › Figure S7.pdf]

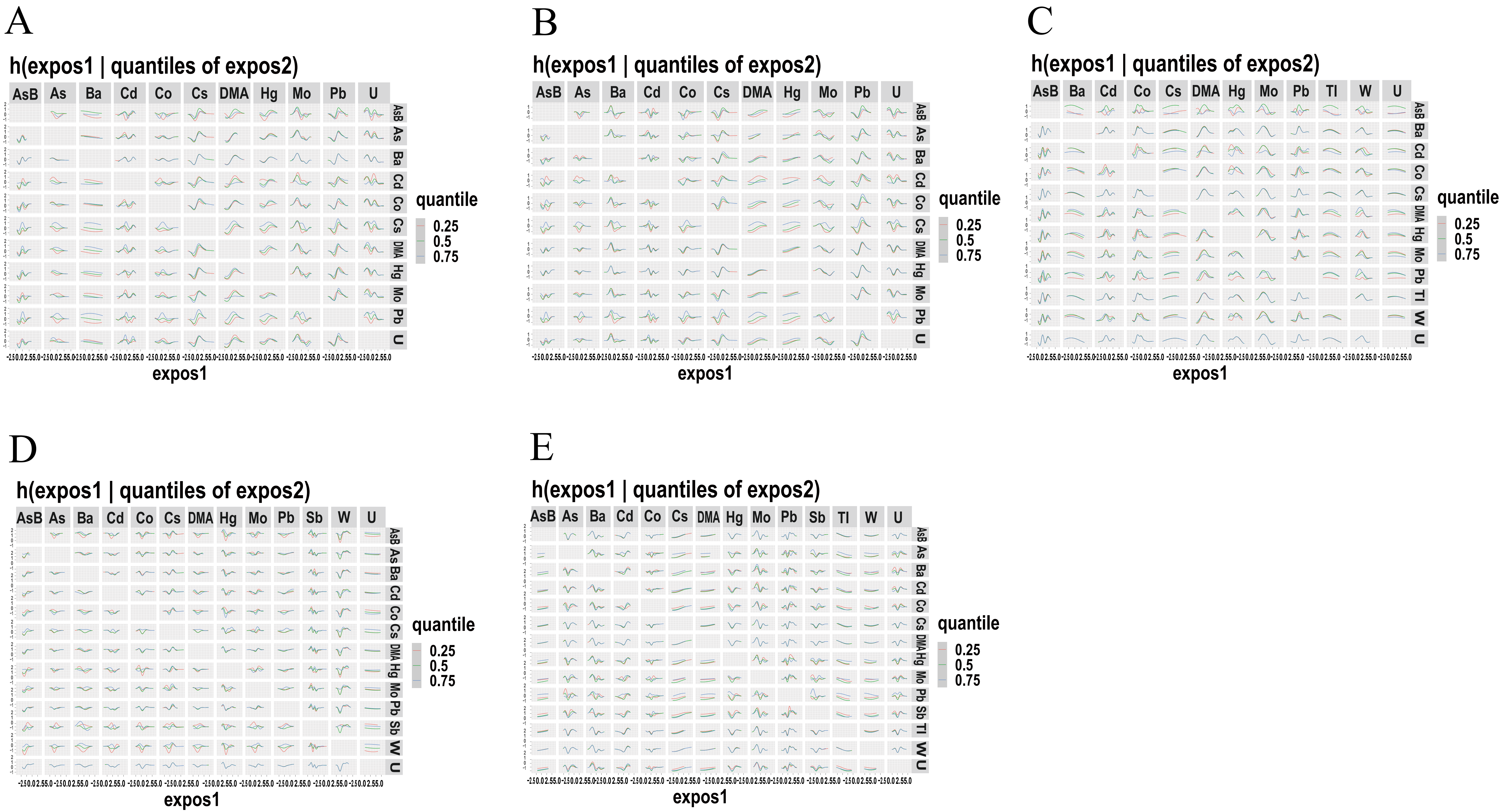
A
h(expos1 | quantiles of expos2)
AsB As Ba Cd Co Cs DMA Hg Mo Pb U
quantile
0.25
0.5
0.75
expos1
B
h(expos1 | quantiles of expos2)
AsB As Ba Cd Co Cs DMA Hg Mo Pb U
quantile
0.25
0.5
0.75
expos1
C
h(expos1 | quantiles of expos2)
AsB Ba Cd Co Cs DMA Hg Mo Pb Tl W U
quantile
0.25
0.5
0.75
expos1
D
h(expos1 | quantiles of expos2)
AsB As Ba Cd Co Cs DMA Hg Mo Pb Sb W U
quantile
0.25
0.5
0.75
expos1
E
h(expos1 | quantiles of expos2)
AsB As Ba Cd Co Cs DMA Hg Mo Pb Sb Tl W U
quantile
0.25
0.5
0.75
expos1
