# Supplementary material for: The Correlation between Metal Mixed Exposure and Lung Function in Different Ages of the Population
Source: Metabolites. 2024 Feb 26;14(3):139. doi: 10.3390/metabo14030139 (PMC10972184; doi:10.3390/metabo14030139)
Supplement: Supplementary file 1 [file metabolites-14-00139-s001.zip › Table S1.pdf]

Table S1. Urinary Metal Distribution in Children and Adolescents of the study population, NHANES 2007–2012 (n =4382).

Metal metabolites	Detection rate n (%)	Mean	LOD	Percentiles				
				P5	P25	P50	P75	P95
Urinary total arsenic	479 (98.79)	8.02	0.26	2.39	3.57	5.13	8.12	21.59
Urinary arsenobetaine	195 (40.63)	2.25	1.19	0.11	0.20	0.42	1.30	11.81
Urinary dimethylarsonic acid	389 (81.04)	3.45	1.91	1.25	1.98	2.78	4.06	8.56
Urinary barium	479 (98.79)	3.57	0.06	0.39	0.93	1.66	2.70	5.46
Urinary cadmium	389 (81.04)	0.09	0.04	0.03	0.05	0.08	0.11	0.18
Urinary cobalt	478 (99.58)	0.49	0.02	0.18	0.28	0.41	0.60	1.12
Urinary cesium	480 (100.00)	3.78	0.09	1.83	2.69	3.56	4.52	6.73
Urinary molybdenum	480 (100.00)	55.87	0.08	19.91	35.68	50.92	68.66	110.83
Urinary lead	460 (95.83)	0.45	0.03	0.13	0.21	0.32	0.46	0.96
Urinary antimony	409 (85.21)	0.08	0.02	0.03	0.04	0.06	0.09	0.16
Urinary thallium	477 (99.38)	0.15	0.02	0.07	0.10	0.14	0.18	0.29
Urinary tungsten	458 (95.42)	0.16	0.02	0.03	0.07	0.11	0.18	0.50
Urinary uranium	461 (96.04)	0.03	0.01	0.00	0.00	0.01	0.01	0.04
Urinary mercury	480 (100.0)	0.45	0.13	0.09	0.15	0.25	0.50	1.35
