# Supplementary material for: The Correlation between Metal Mixed Exposure and Lung Function in Different Ages of the Population
Source: Metabolites. 2024 Feb 26;14(3):139. doi: 10.3390/metabo14030139 (PMC10972184; doi:10.3390/metabo14030139)
Supplement: Supplementary file 1 [file metabolites-14-00139-s001.zip › Table S2.pdf]

Table S2. Urinary Metal Distribution in Adults of the study population, NHANES 2007–2012 (n =4382).

**Metal metabolites**	**Detection rate n (%)**	**Mean**	**LOD**	**Percentiles**				
				**P5**	**P25**	**P50**	**P75**	**P95**
Urinary total arsenic	2840 (98.44)	17.56	0.26	2.30	4.33	7.31	14.99	58.20
Urinary arsenobetaine	1685 (58.41)	8.82	1.19	0.16	0.46	1.43	5.44	34.25
Urinary dimethylarsonic acid	2351 (81.49)	5.29	1.91	1.34	2.29	3.61	6.04	13.83
Urinary barium	2871 (99.52)	1.99	0.06	0.31	0.74	1.32	2.32	5.61
Urinary cadmium	2640 (91.51)	0.29	0.04	0.05	0.11	0.19	0.34	0.83
Urinary cobalt	2868 (99.41)	0.44	0.02	0.13	0.21	0.31	0.48	1.16
Urinary cesium	2885 (100.0)	4.61	0.09	1.87	2.93	3.97	5.57	9.23
Urinary molybdenum	2884 (99.97)	48.54	0.08	14.67	27.38	39.56	58.24	108.55
Urinary lead	2775 (96.19)	0.60	0.03	0.15	0.27	0.42	0.66	1.45
Urinary antimony	2029 (70.33)	0.08	0.02	0.02	0.04	0.05	0.08	0.19
Urinary thallium	2863 (99.24)	0.17	0.02	0.07	0.11	0.15	0.21	0.37
Urinary tungsten	2527 (87.59)	0.12	0.02	0.02	0.04	0.07	0.13	0.35
Urinary uranium	2464 (85.41)	0.01	0.01	0.00	0.00	0.01	0.01	0.03
Urinary mercury	2885 (100.0)	0.66	0.13	0.09	0.20	0.39	0.75	2.07
