# Supplementary material for: The Correlation between Metal Mixed Exposure and Lung Function in Different Ages of the Population
Source: Metabolites. 2024 Feb 26;14(3):139. doi: 10.3390/metabo14030139 (PMC10972184; doi:10.3390/metabo14030139)
Supplement: Supplementary file 1 [file metabolites-14-00139-s001.zip › Table S3.pdf]

Table S3. Urinary Metal Distribution in the elderly of the study population, NHANES 2007–2012 (n =4382).

Metal metabolites	Detection rate n (%)	Mean	LOD	Percentiles				
				P5	P25	P50	P75	P95
Urinary total arsenic	1007 (99.02)	20.93	0.26	2.84	5.06	9.34	18.95	78.01
Urinary arsenobetaine	657 (57.89)	10.98	1.19	0.21	0.65	2.10	8.59	44.78
Urinary dimethylarsonic acid	837 (64.60)	6.07	1.91	1.68	2.76	4.18	6.84	15.80
Urinary barium	1011 (99.41)	2.30	0.06	0.26	0.67	1.33	2.63	6.84
Urinary cadmium	991 (97.44)	0.48	0.04	0.13	0.24	0.38	0.59	1.20
Urinary cobalt	1012 (99.51)	0.49	0.02	0.14	0.23	0.33	0.49	1.12
Urinary cesium	1017 (100.0)	5.59	0.09	2.21	3.60	4.75	6.59	11.01
Urinary molybdenum	1016 (99.90)	54.52	0.08	14.81	28.16	41.40	65.07	138.54
Urinary lead	1007 (99.02)	0.85	0.03	0.26	0.45	0.66	0.97	2.00
Urinary antimony	657 (57.89)	0.08	0.02	0.02	0.04	0.06	0.09	0.19
Urinary thallium	1016 (99.90)	0.18	0.02	0.06	0.11	0.15	0.22	0.39
Urinary tungsten	861 (84.66)	0.11	0.02	0.02	0.04	0.07	0.13	0.32
Urinary uranium	862 (84.76)	0.01	0.01	0.00	0.00	0.01	0.01	0.04
Urinary mercury	1017 (100.0)	0.72	0.13	0.10	0.23	0.43	0.85	2.19
