# Supplementary material for: The Correlation between Metal Mixed Exposure and Lung Function in Different Ages of the Population
Source: Metabolites. 2024 Feb 26;14(3):139. doi: 10.3390/metabo14030139 (PMC10972184; doi:10.3390/metabo14030139)
Supplement: Supplementary file 1 [file metabolites-14-00139-s001.zip › Table S4.pdf]

Table S4. Relationship between metal and lung function of the study population, NHANES 2007–2012 (n = 4382)

	**FEV1(mL)** **β (95%CI)**	**FVC(mL)** **β (95%CI)**	**FEF25–75%(mL/s)** **β (95%CI)**	**FET(s)** **β (95%CI)**	**PEF(mL/s)** **β (95%CI)**
**Children and adolescents**					
**Group (6-18)**					
Urinary arsenobetaine	0.004 (-0.046, 0.054)	0.017 (-0.029, 0.064)	-0.012 (-0.067, 0.043)	-0.047 (-0.154, 0.060)	0.006 (-0.047, 0.059)
Urinary dimethylarsonic acid	-0.074 (-0.195, 0.047)	-0.069 (-0.181, 0.044)	0.006 (-0.137, 0.149)	0.185 (-0.077, 0.447)	-0.030 (-0.162, 0.101)
Urinary cadmium	-0.052 (-0.125, 0.021)	-0.048 (-0.116, 0.019)	-0.041 (-0.160, 0.078)	-0.14 (-0.297, 0.017))	-0.030 (-0.108, 0.048)
Urinary cobalt	-0.029 (-0.118, 0.06)	-0.053 (-0.135, 0.030)	0.024 (-0.121, 0.169)	-0.097 (-0.287, 0.094)	-0.035 (-0.129, 0.060)
Urinary molybdenum	-0.038 (-0.127, 0.051)	-0.006 (-0.090, 0.079)	-0.019 (-0.183, 0.144)	0.106 (-0.108, 0.320)	0.006 (-0.101, 0.112)
Urinary lead	**-0.190 (-0.266, -0.114)** [a]	**-0.184 (-0.254, -0.114)** [a]	**-0.167 (-0.291, -0.043)** [a]	0.032 (-0.135, 0.200)	**-0.157 (-0.240, -0.073)** [a]
Urinary uranium	-0.024 (-0.074, 0.026)	-0.032 (-0.078, 0.014)	-0.006 (-0.092, 0.079)	-0.02 (-0.132, 0.092)	-0.038 (-0.094, 0.018)
Urinary mercury	0.018 (-0.035, 0.071)	-0.004 (-0.053, 0.045)	0.083 (-0.006, 0.172)	-0.054 (-0.170, 0.062)	0.039 (-0.019, 0.097)
Urinary barium	**0.086 (0.030, 0.142)** [a]	**0.092 (0.041, 0.144)** [a]	0.062 (-0.028, 0.153)	-0.001 (-0.120, 0.117)	0.049 (-0.010, 0.109)
Urinary total arsenic	0.046 (-0.085, 0.177)	0.023 (-0.098, 0.144)	-	-0.005 (-0.283, 0.272)	0.004 (-0.135, 0.143)
Urinary cesium	-	-0.021 (-0.130, 0.088)	0.044 (-0.193, 0.281)	0.123 (-0.127, 0.373)	0.001 (-0.153, 0.154)
Urinary tungsten	-	-	-0.077 (-0.183, 0.029)	0.001 (-0.137, 0.139)	-0.024 (-0.093, 0.045)
Urinary antimony	-	-	-	-0.039 (-0.210, 0.132)	0.016 (-0.069, 0.101)
Urinary thallium	-	-	**-0.237 (-0.431, -0.043)** [a]	-	**-0.131 (-0.258, -0.005)** [a]
**Adult group (19-59)**					
Urinary arsenobetaine	0.002 (-0.018, 0.022)	0.008 (-0.011, 0.027)	0.083 (0.027, 0.139)	0.009 (-0.025, 0.044)	-0.009 (-0.031, 0.013)
Urinary dimethylarsonic acid	0.006 (-0.031, 0.044)	-0.019 (-0.054, 0.017)	**0.002 (-0.039, 0.043)** [a]	-0.020 (-0.084, 0.044)	-0.003 (-0.044, 0.038)
Urinary cadmium	**-0.174 (-0.197, -0.151)** [a]	**-0.113 (-0.134, -0.091)** [a]	**-0.322 (-0.368, -0.276)** [a]	**0.176 (0.136, 0.215)** [a]	**-0.109 (-0.134, -0.084)** [a]
Urinary cobalt	0.021 (-0.010, 0.052)	0.013 (-0.016, 0.043)	**0.065 (0.004, 0.127)** [a]	-0.021 (-0.073, 0.032)	0.015 (-0.019, 0.049)

Urinary molybdenum	0.006 (-0.021, 0.033)	-0.006 (-0.031, 0.020)	0.032 (-0.025, 0.089)	**-0.074 (-0.123, -0.025)** [a]	0.009 (-0.023, 0.040)
Urinary lead	**-0.100 (-0.127, -0.073)** [a]	**-0.078 (-0.104, -0.053)** [a]	**-0.134 (-0.187, -0.08)** [a]	**0.071 (0.025, 0.117)** [a]	**-0.057 (-0.086, -0.027)** [a]
Urinary uranium	0.002 (-0.018, 0.022)	0.011 (-0.008, 0.029)	-0.026 (-0.067, 0.015)	0.024 (-0.011, 0.059)	-0.012 (-0.035, 0.010)
Urinary mercury	0.001 (-0.018, 0.019)	0.008 (-0.009, 0.026)	-0.016 (-0.054, 0.021)	**0.043 (0.011, 0.075)** [a]	**0.030 (0.009, 0.051)** [a]
Urinary barium	0.017 (-0.004, 0.037)	**0.021 (0.001, 0.040)** [a]	0.083 (0.027, 0.139)	-0.010 (-0.046, 0.025)	0.010 (-0.012, 0.033)
Urinary total arsenic	-0.027 (-0.069, 0.016)	-0.032 (-0.071, 0.008)	-	-0.019 (-0.091, 0.053)	-0.003 (-0.050, 0.043)
Urinary cesium	-	0.016 (-0.021, 0.052)	**-0.178 (-0.268, -0.088)** [a]	**0.134 (0.068, 0.201)** [a]	-0.006 (-0.030, 0.018)
Urinary tungsten	-	-	-0.004 (-0.046, 0.039)	0.022 (-0.015, 0.058)	0.016 (-0.013, 0.044)
Urinary antimony	-	-	-	**-0.071 (-0.115, -0.026)** [a]	0.005 (-0.040, 0.049)
Urinary thallium	-	-	0.072 (-0.009, 0.153)	-	-0.009 (-0.031, 0.013)
**Elderly peoples (⩾60)**					
Urinary arsenobetaine	0.023 (-0.025, 0.071)	0.005 (-0.037, 0.047)	**0.066 (0.009, 0.124)** [a]	-0.026 (-0.097, 0.046)	0.028 (-0.029, 0.085)
Urinary dimethylarsonic acid	-0.066 (-0.151, 0.019)	**-0.075 (-0.149, -0.001)** [a]	-0.059 (-0.201, 0.083)	-0.073 (-0.201, 0.055)	-0.038 (-0.139, 0.064)
Urinary cadmium	**-0.132 (-0.189, -0.075)** [a]	**-0.072 (-0.122, -0.023)** [a]	**-0.337 (-0.465, -0.209)** [a]	0.025 (-0.061, 0.110)	**-0.123 (-0.190, -0.055)** [a]
Urinary cobalt	**-0.085 (-0.147, -0.023)** [a]	-0.041 (-0.094, 0.013)	**-0.255 (-0.394, -0.116)** [a]	0.053 (-0.04, 0.145)	**-0.084 (-0.157, -0.011)** [a]
Urinary molybdenum	-0.053 (-0.110, 0.004)	**-0.079 (-0.129, -0.030)** [a]	-0.018 (-0.153, 0.117)	-0.06 (-0.149, 0.030)	-0.061 (-0.133, 0.010)
Urinary lead	0.022 (-0.042, 0.086)	0.006 (-0.050, 0.061)	0.015 (-0.129, 0.159)	0.007 (-0.089, 0.104)	0.03 (-0.046, 0.106)
Urinary uranium	**-0.062 (-0.104, -0.019)** [a]	-0.030 (-0.067, 0.006)	**-0.138 (-0.236, -0.041)** [a]	0.025 (-0.040, 0.091)	**-0.053 (-0.105, -0.001)** [a]
Urinary mercury	**0.061 (0.018, 0.104)** [a]	**0.043 (0.006, 0.081)** [a]	0.068 (-0.029, 0.166)	-0.026 (-0.091, 0.038)	0.026 (-0.025, 0.078)
Urinary barium	**0.099 (0.058, 0.140)** [a]	**0.076 (0.040, 0.112)** [a]	**0.147 (0.054, 0.240)** [a]	0.003 (-0.059, 0.065)	**0.084 (0.035, 0.133)** [a]
Urinary total arsenic	-0.008 (-0.107, 0.091)	-0.004 (-0.090, 0.081)	-	0.013 (-0.134, 0.160)	-0.025 (-0.141, 0.091)
Urinary cesium	-	**0.119 (0.043, 0.195)** [a]	0.043 (-0.170, 0.257)	**0.140 (0.009, 0.271)** [a]	0.102 (-0.010, 0.215)
Urinary tungsten	-	-	0.003 (-0.108, 0.115)	0.037 (-0.038, 0.111)	0.031 (-0.028, 0.090)
Urinary antimony	-	-	-	-0.005 (-0.094, 0.084)	-0.040 (-0.110, 0.031)
Urinary thallium	-	-	-0.008 (-0.188, 0.173)	-	-0.022 (-0.118, 0.073)

Notes: FEV1, forced expiratory volume in 1s; FVC, forced vital capacity; FEF25–75%, forced expiratory fow between 25 and 75% of FVC; PEF, peak expiratory fow rate. FET, forced expiratory time. Adjusted for age, sex, race/ethnicity, education level, marital status, ratio of family income to poverty, BMI, physical activity level, alcohol status and smoking status.

[a] $P < 0.05$.
