# Supplementary material for: The Correlation between Metal Mixed Exposure and Lung Function in Different Ages of the Population
Source: Metabolites. 2024 Feb 26;14(3):139. doi: 10.3390/metabo14030139 (PMC10972184; doi:10.3390/metabo14030139)
Supplement: Supplementary file 1 [file metabolites-14-00139-s001.zip › Table S5.pdf]

**Table S5.** Relationship between metal and lung function of the study population, NHANES 2007–2012 (n =4382).

| | FEV1(mL)<br>β (95%CI) | FVC(mL)<br>β (95%CI) | FEF25–75%(mL/s)<br>β (95%CI) | FET(s)<br>β (95%CI) | PEF(mL/s)<br>β (95%CI) |
|---|---|---|---|---|---|
| **Children and adolescents Group (6-18)** | | | | | |
| Urinary arsenobetaine | 0.011 (-0.046, 0.067) | 0.022 (-0.032, 0.077) | -0.015 (-0.071, 0.041) | -0.030 (-0.135, 0.075) | 0.014 (-0.043, 0.072) |
| Urinary dimethylarsonic acid | -0.067 (-0.203, 0.069) | -0.078 (-0.211, 0.055) | 0.015 (-0.130, 0.161) | 0.171 (-0.088, 0.430) | -0.021 (-0.163, 0.121) |
| Urinary cadmium | **-0.090 (-0.172, -0.007)**[a] | **-0.088 (-0.167, -0.008)**[a] | -0.072 (-0.194, 0.050) | -0.132 (-0.287, 0.024) | -0.060 (-0.145, 0.024) |
| Urinary cobalt | **-0.165 (-0.259, -0.071)**[a] | **-0.225 (-0.317, -0.134)**[a] | -0.051 (-0.192, 0.091) | -0.163 (-0.341, 0.015) | **-0.177 (-0.274, -0.081)**[a] |
| Urinary molybdenum | 0.002 (-0.098, 0.102) | 0.034 (-0.066, 0.134) | -0.003 (-0.172, 0.166) | 0.110 (-0.102, 0.322) | 0.039 (-0.077, 0.155) |
| Urinary lead | **-0.188 (-0.272, -0.103)**[a] | **-0.180 (-0.262, -0.098)**[a] | **-0.176 (-0.303, -0.050)**[a] | 0.037 (-0.127, 0.202) | **-0.149 (-0.239, -0.060)**[a] |
| Urinary uranium | 0.013 (-0.042, 0.069) | 0.005 (-0.049, 0.059) | 0.034 (-0.054, 0.121) | -0.014 (-0.124, 0.097) | -0.006 (-0.066, 0.054) |
| Urinary mercury | -0.002 (-0.062, 0.058) | -0.030 (-0.088, 0.028) | 0.068 (-0.023, 0.160) | -0.065 (-0.18, 0.050) | 0.017 (-0.046, 0.079) |
| Urinary barium | **0.102 (0.039, 0.165)**[a] | **0.115 (0.054, 0.176)**[a] | 0.070 (-0.023, 0.163) | 0.014 (-0.104, 0.132) | **0.071 (0.007, 0.136)**[a] |
| Urinary total arsenic | 0.020 (-0.128, 0.168) | 0.003 (-0.140, 0.146) | - | -0.025 (-0.301, 0.251) | 0.023 (-0.141, 0.187) |
| Urinary cesium | - | 0.016 (-0.112, 0.145) | 0.103 (-0.138, 0.343) | 0.105 (-0.143, 0.353) | -0.030 (-0.105, 0.045) |
| Urinary tungsten | - | - | -0.081 (-0.191, 0.029) | -0.006 (-0.144, 0.132) | -0.017 (-0.11, 0.076) |
| Urinary antimony | - | - | - | -0.059 (-0.229, 0.111) | -0.019 (-0.170, 0.133) |
| Urinary thallium | - | - | **-0.261 (-0.457, -0.064)**[a] | - | -0.129 (-0.264, 0.007) |
| **Adult group (19-59)** | | | | | |
| Urinary arsenobetaine | -0.022 (-0.044, 0.001) | -0.014 (-0.037, 0.009) | **-0.026 (-0.048, -0.003)**[a] | 0.006 (-0.028, 0.04) | -0.001 (-0.026, 0.023) |
| Urinary dimethylarsonic acid | -0.009 (-0.053, 0.034) | -0.04 (-0.084, 0.005) | **0.122 (0.065, 0.179)**[a] | -0.04 (-0.105, 0.026) | 0.026 (-0.022, 0.073) |
| Urinary cadmium | **-0.271 (-0.297, -0.245)**[a] | **-0.205 (-0.232, -0.179)**[a] | **-0.426 (-0.469, -0.382)**[a] | **0.142 (0.103, 0.180)**[a] | **-0.212 (-0.240, -0.184)**[a] |
| Urinary cobalt | **-0.181 (-0.215, -0.147)**[a] | **-0.219 (-0.254, -0.184)**[a] | -0.037 (-0.095, 0.021) | **-0.182 (-0.233, -0.131)**[a] | **-0.209 (-0.246, -0.172)**[a] |
| Urinary molybdenum | -0.000 (-0.033, 0.033) | -0.016 (-0.049, 0.018) | 0.056 (-0.002, 0.115) | **-0.084 (-0.135, -0.032)**[a] | 0.004 (-0.033, 0.041) |

| | | | | | |
|---|---|---|---|---|---|
| Urinary lead | -0.012 (-0.044, 0.019) | 0.026 (-0.006, 0.058) | **-0.077 (-0.130, -0.025)[a]** | **0.129 (0.082, 0.176)[a]** | 0.015 (-0.019, 0.049) |
| Urinary uranium | 0.004 (-0.020, 0.028) | 0.013 (-0.011, 0.037) | -0.026 (-0.067, 0.015) | 0.014 (-0.023, 0.051) | -0.024 (-0.051, 0.003) |
| Urinary mercury | -0.022 (-0.044, 0.000) | -0.017 (-0.04, 0.005) | -0.019 (-0.057, 0.018) | 0.024 (-0.009, 0.057) | 0.009 (-0.015, 0.033) |
| Urinary barium | **0.040 (0.015, 0.065)[a]** | **0.051 (0.026, 0.077)[a]** | 0.008 (-0.034, 0.050) | 0.014 (-0.023, 0.051) | **0.038 (0.011, 0.065)[a]** |
| Urinary total arsenic | 0.025 (-0.024, 0.074) | 0.021 (-0.029, 0.070) | - | -0.009 (-0.082, 0.064) | -0.003 (-0.056, 0.050) |
| Urinary cesium | - | -0.019 (-0.066, 0.028) | **-0.152 (-0.244, -0.060)[a]** | **0.113 (0.044, 0.182)[a]** | -0.023 (-0.082, 0.036) |
| Urinary tungsten | - | - | -0.016 (-0.06, 0.027) | 0.012 (-0.027, 0.051) | -0.015 (-0.043, 0.013) |
| Urinary antimony | - | - | - | **-0.094 (-0.141, -0.048)[a]** | -0.008 (-0.042, 0.026) |
| Urinary thallium | - | - | 0.017 (-0.064, 0.099) | - | -0.03 (-0.082, 0.022) |
| **Elderly peoples (⩾60)** | | | | | |
| Urinary arsenobetaine | 0.010 (-0.043, 0.063) | 0.004 (-0.048, 0.055) | 0.053 (-0.004, 0.109) | -0.015 (-0.086, 0.056) | 0.036 (-0.025, 0.097) |
| Urinary dimethylarsonic acid | **-0.128 (-0.224, -0.032)[a]** | **-0.138 (-0.233, -0.044)[a]** | -0.061 (-0.205, 0.082) | -0.106 (-0.236, 0.024) | -0.081 (-0.193, 0.031) |
| Urinary cadmium | **-0.246 (-0.307, -0.184)[a]** | **-0.183 (-0.243, -0.123)[a]** | **-0.446 (-0.568, -0.325)[a]** | -0.024 (-0.107, 0.059) | **-0.267 (-0.338, -0.196)[a]** |
| Urinary cobalt | **-0.113 (-0.184, -0.043)[a]** | **-0.079 (-0.149, -0.010)[a]** | **-0.253 (-0.393, -0.114)[a]** | 0.034 (-0.061, 0.130) | **-0.114 (-0.196, -0.033)[a]** |
| Urinary molybdenum | -0.050 (-0.116, 0.016) | **-0.078 (-0.142, -0.014)[a]** | 0.000 (-0.135, 0.136) | -0.062 (-0.155, 0.030) | -0.040 (-0.119, 0.040) |
| Urinary lead | **0.092 (0.020, 0.163)[a]** | **0.101 (0.031, 0.171)[a]** | 0.016 (-0.125, 0.156) | 0.073 (-0.024, 0.171) | 0.085 (0.002, 0.169) |
| Urinary uranium | **-0.123 (-0.171, -0.076)[a]** | **-0.114 (-0.160, -0.068)[a]** | **-0.131 (-0.227, -0.035)[a]** | -0.018 (-0.085, 0.048) | **-0.112 (-0.168, -0.055)[a]** |
| Urinary mercury | **0.063 (0.017, 0.110)[a]** | 0.029 (-0.018, 0.075) | **0.115 (0.021, 0.209)[a]** | -0.035 (-0.099, 0.029) | 0.047 (-0.008, 0.103) |
| Urinary barium | **0.076 (0.029, 0.123)[a]** | 0.046 (-0.001, 0.092) | **0.148 (0.055, 0.242)[a]** | -0.009 (-0.074, 0.055) | 0.079 (0.023, 0.134) |
| Urinary total arsenic | 0.045 (-0.066, 0.155) | 0.038 (-0.070, 0.145) | - | 0.020 (-0.128, 0.167) | 0.006 (-0.121, 0.132) |
| Urinary cesium | - | 0.088 (-0.009, 0.185) | 0.146 (-0.065, 0.357) | 0.110 (-0.024, 0.244) | **0.156 (0.032, 0.279)[a]** |
| Urinary tungsten | - | - | -0.008 (-0.119, 0.102) | 0.027 (-0.049, 0.103) | 0.004 (-0.061, 0.070) |
| Urinary antimony | - | - | - | -0.040 (-0.132, 0.051) | **-0.098 (-0.176, -0.019)[a]** |

| | | | | | |
|---|---|---|---|---|---|
| Urinary thallium | - | - | -0.056 (-0.233, 0.122) | - | **-0.131 (-0.235, -0.026)**[a] |

Notes: FEV1, forced expiratory volume in 1s; FVC, forced vital capacity; $FEF_{25-75\%}$, forced expiratory fow between 25 and 75% of FVC; PEF, peak expiratory fow rate.

[a] $P < 0.05$
