# Supplementary figures and images for: The Correlation between Metal Mixed Exposure and Lung Function in Different Ages of the Population
Source: Metabolites. 2024 Feb 26;14(3):139. doi: 10.3390/metabo14030139 (PMC10972184; doi:10.3390/metabo14030139)

## Children and Adolescents

A

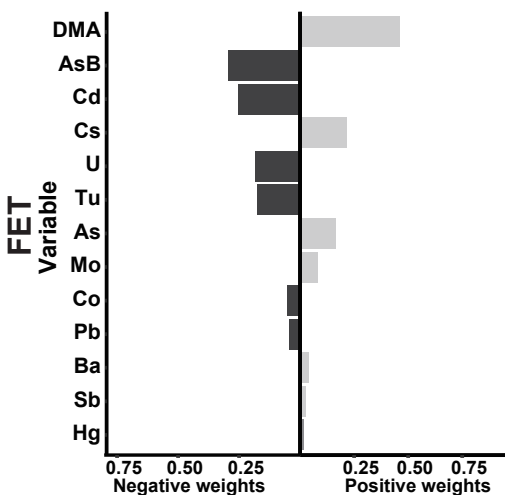

C

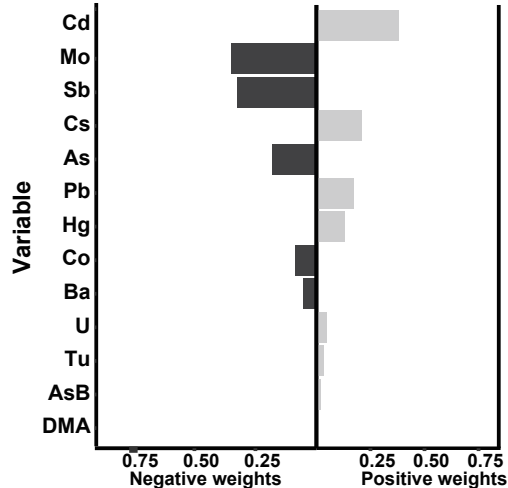

## Elderly

E

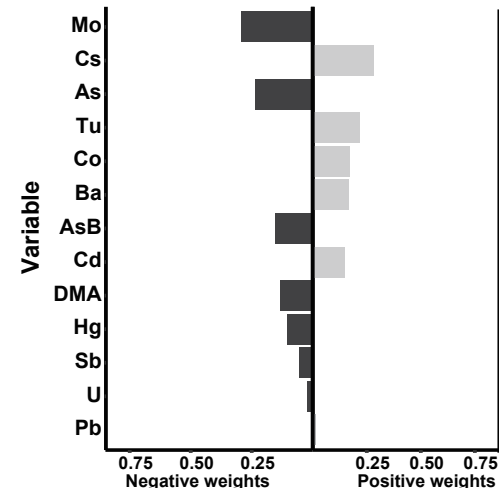

B

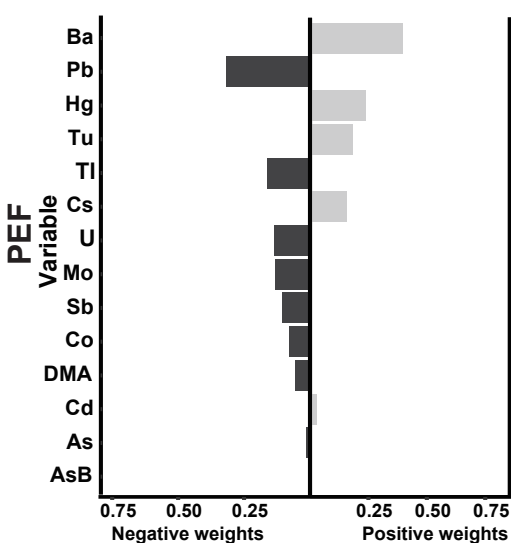

D

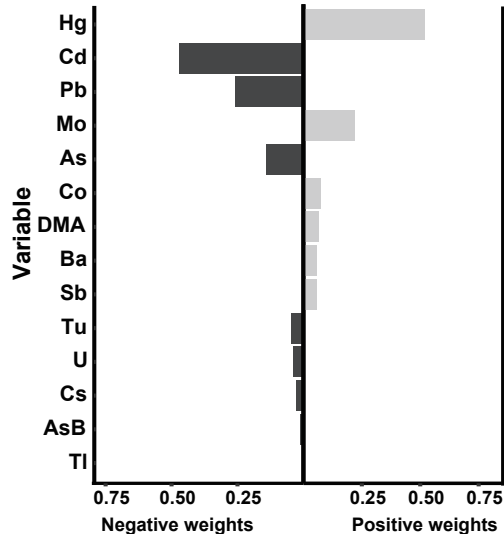

F

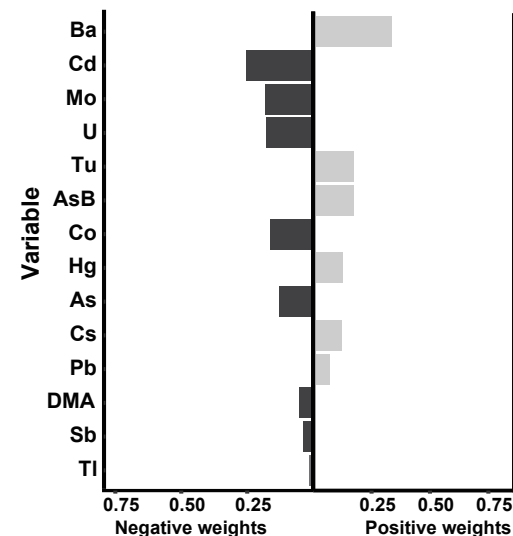

Supplement: Supplementary file 1 [file metabolites-14-00139-s001.zip › Figure S1.pdf]

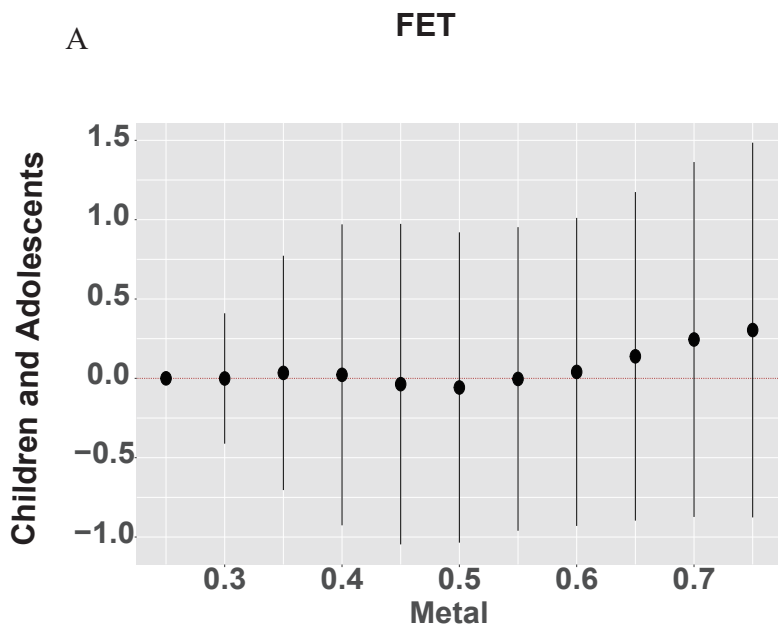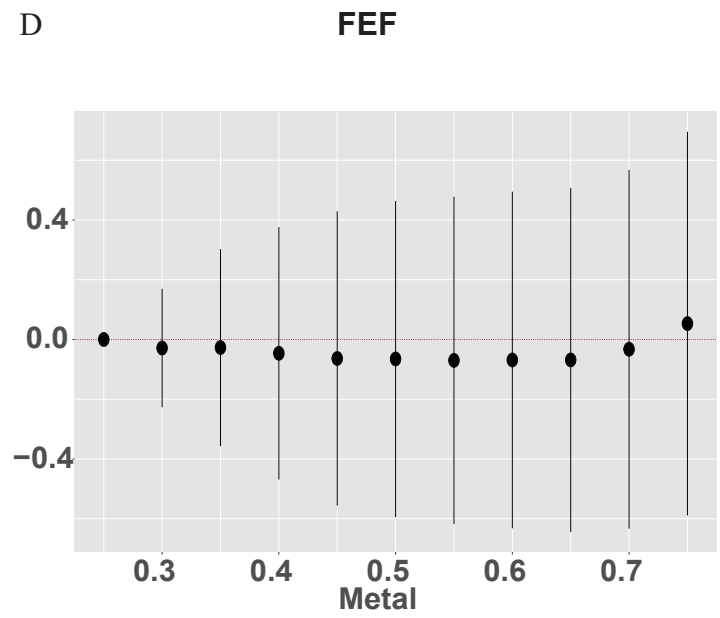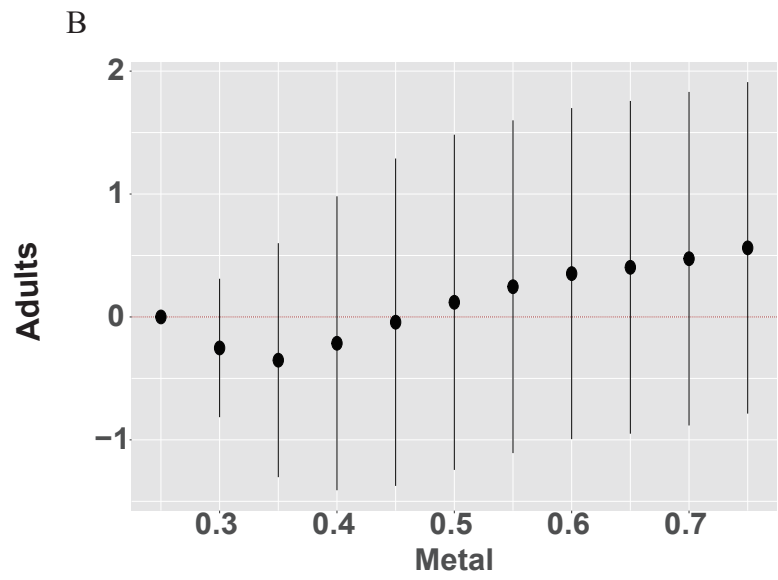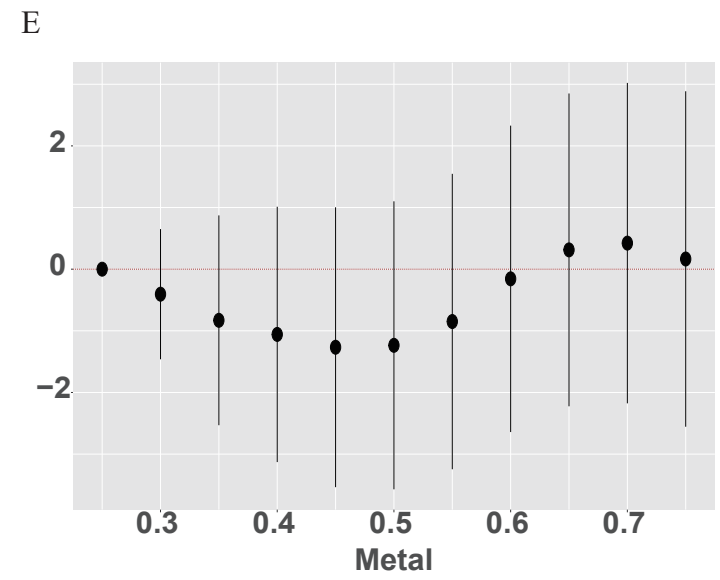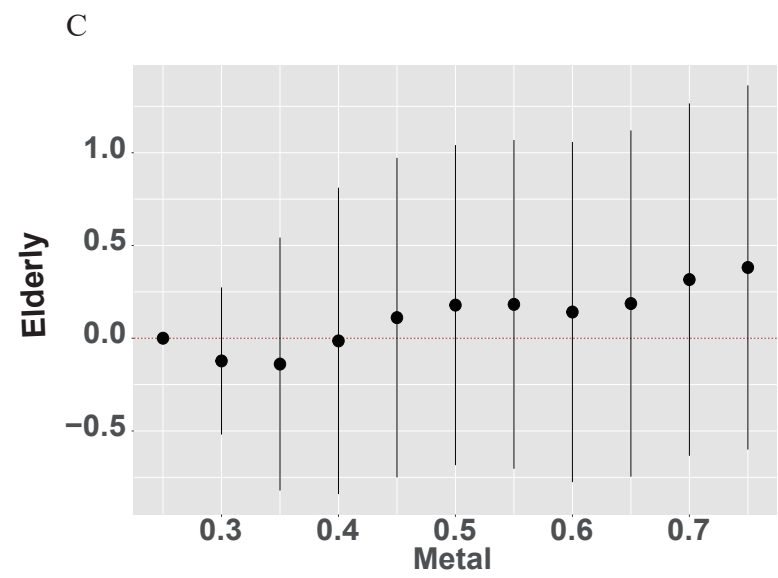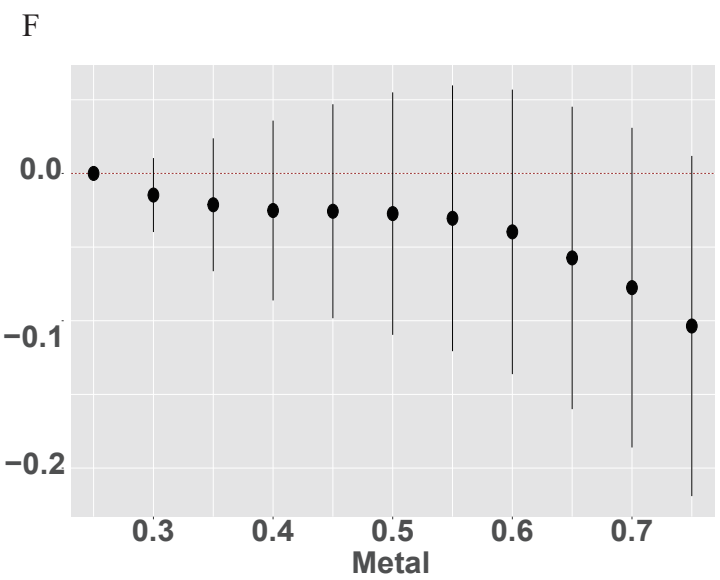

Supplement: Supplementary file 1 [file metabolites-14-00139-s001.zip › Figure S2.pdf]

A

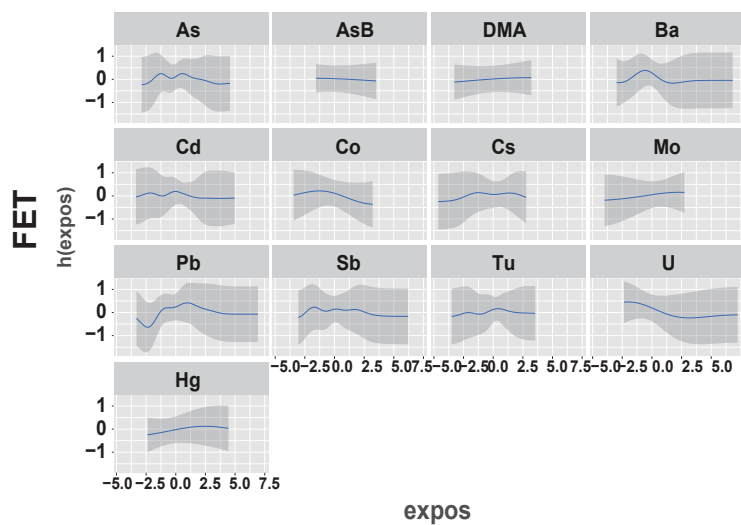

B

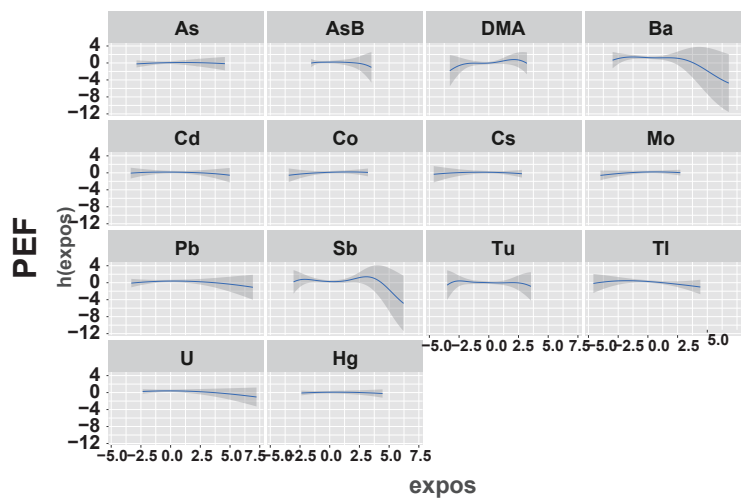

C

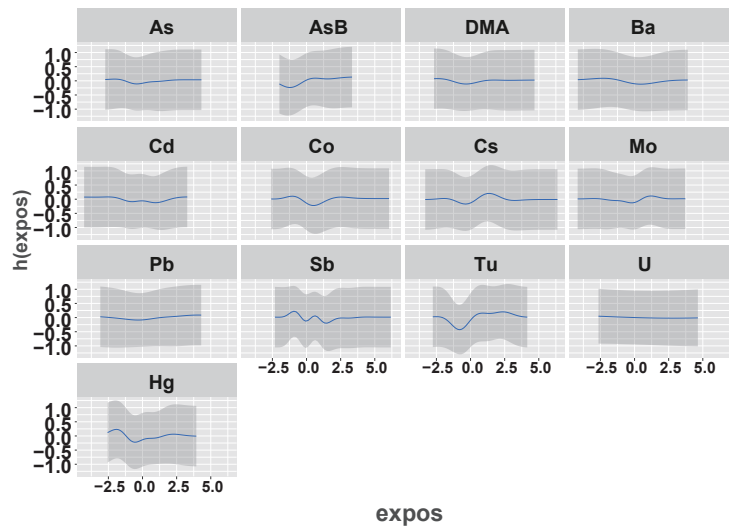

D

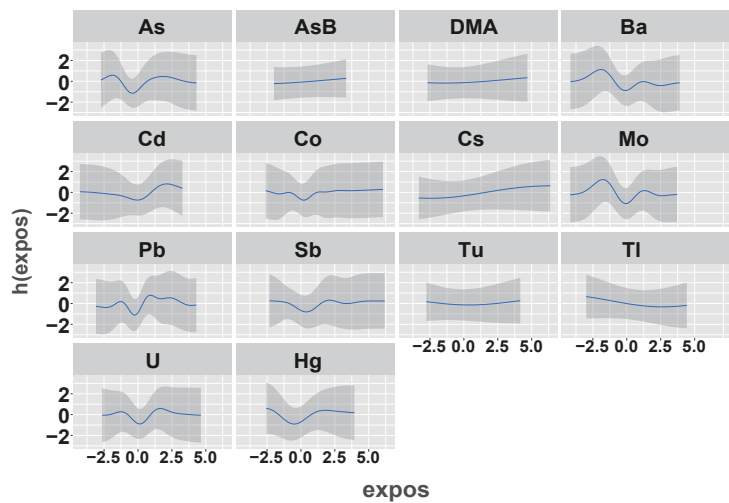

E

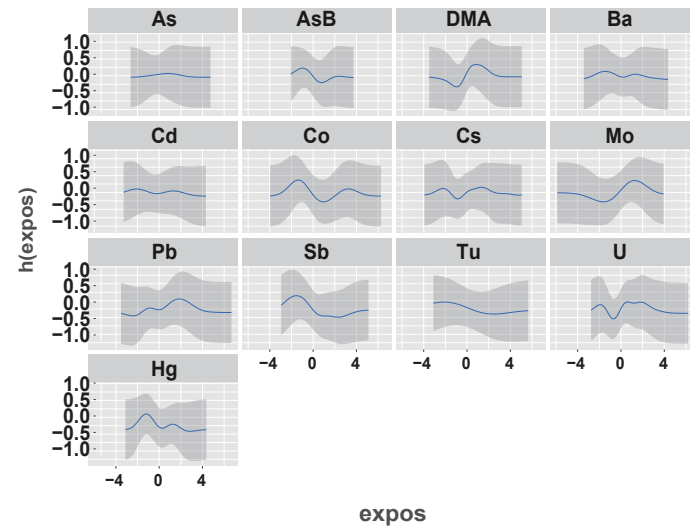

F

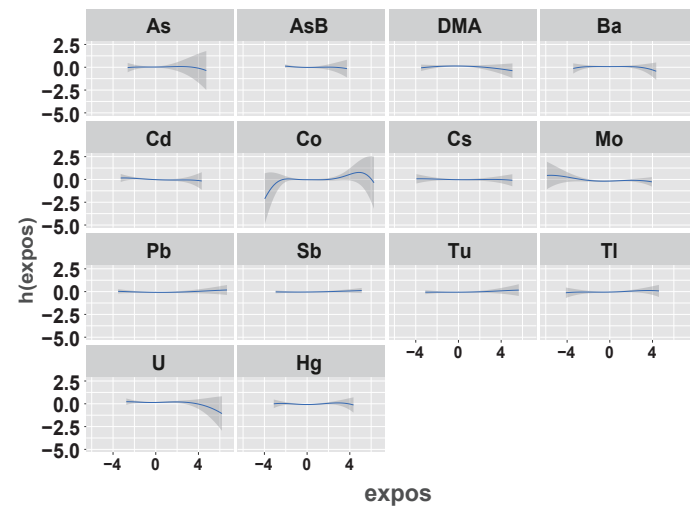

Supplement: Supplementary file 1 [file metabolites-14-00139-s001.zip › Figure S3.pdf]

Children and Adolescents

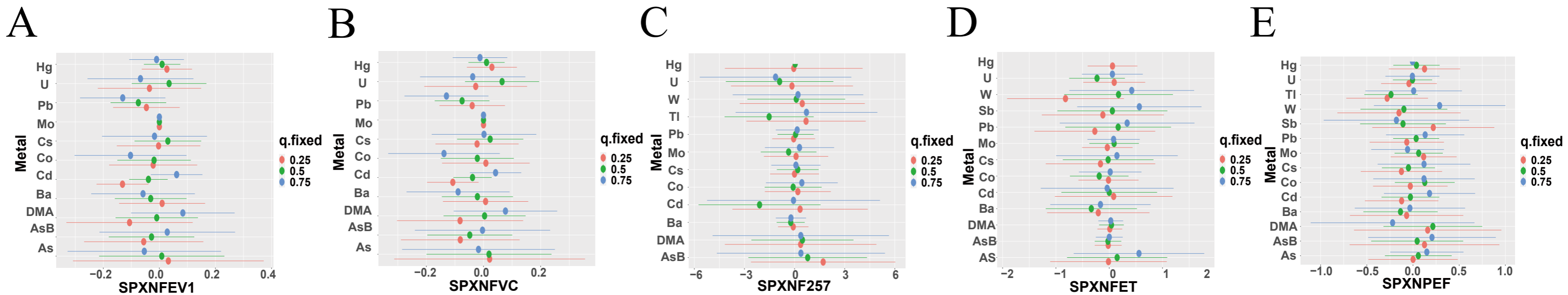

Adults

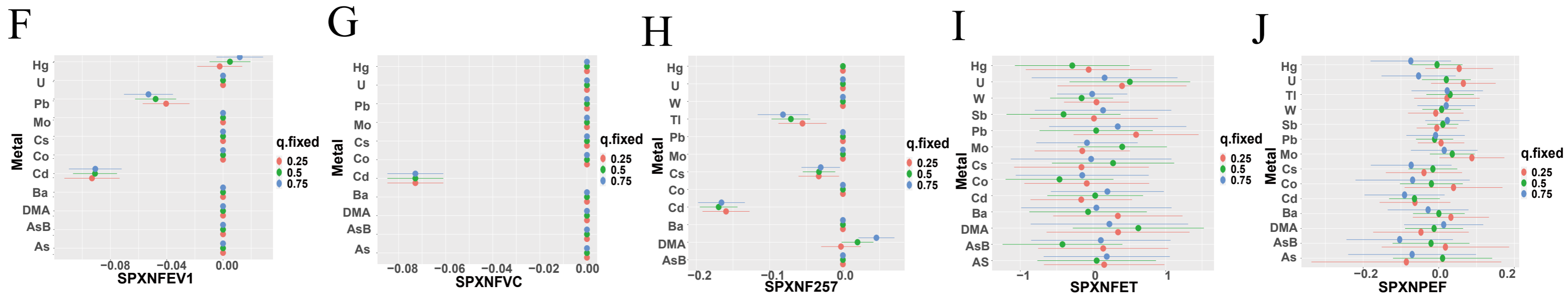

Elderly

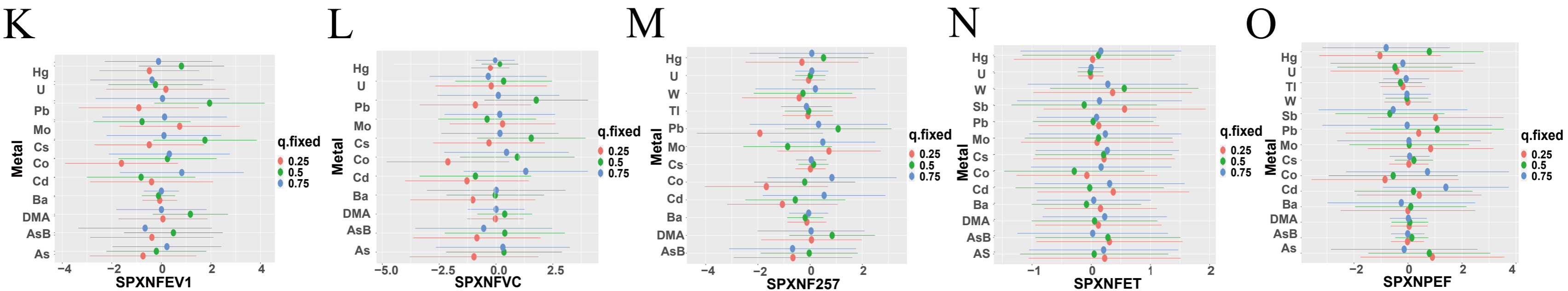

Supplement: Supplementary file 1 [file metabolites-14-00139-s001.zip › Figure S4.pdf]

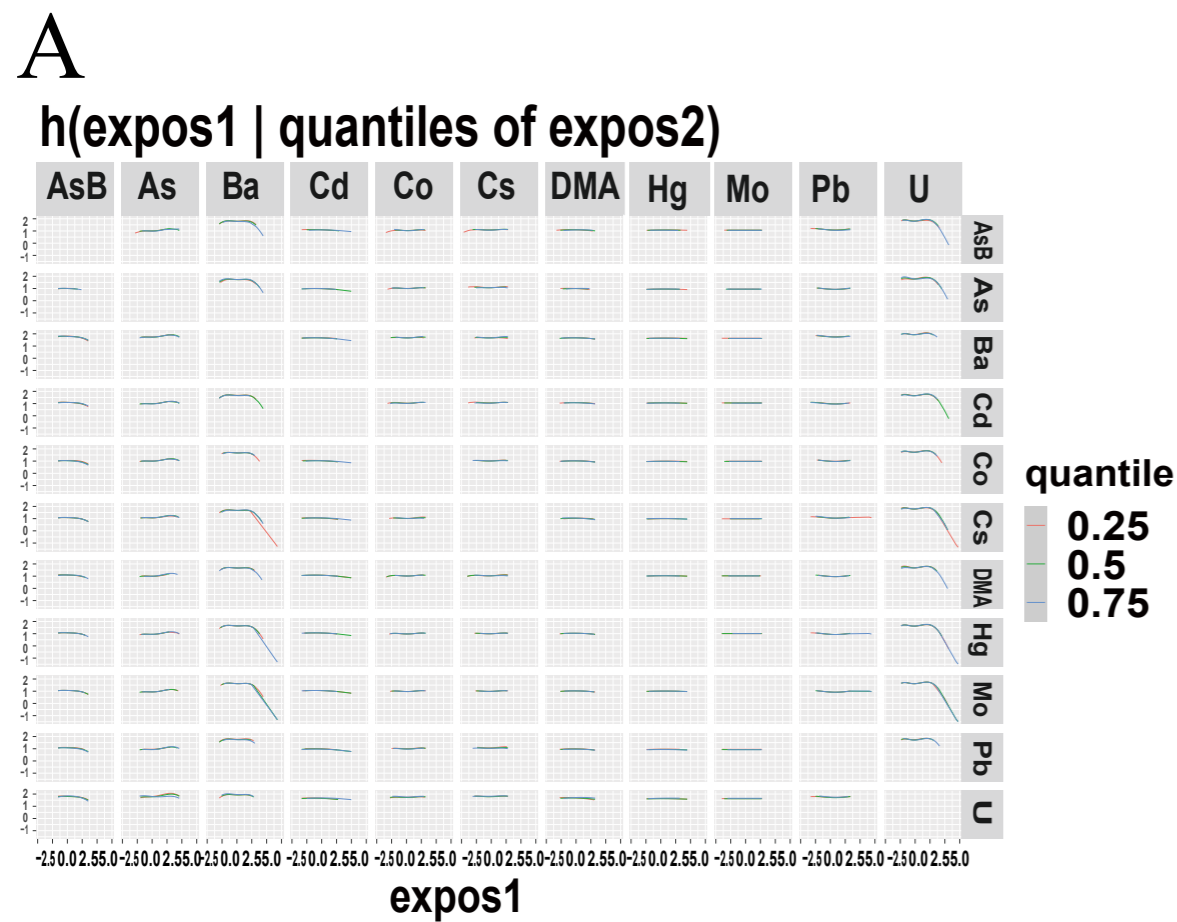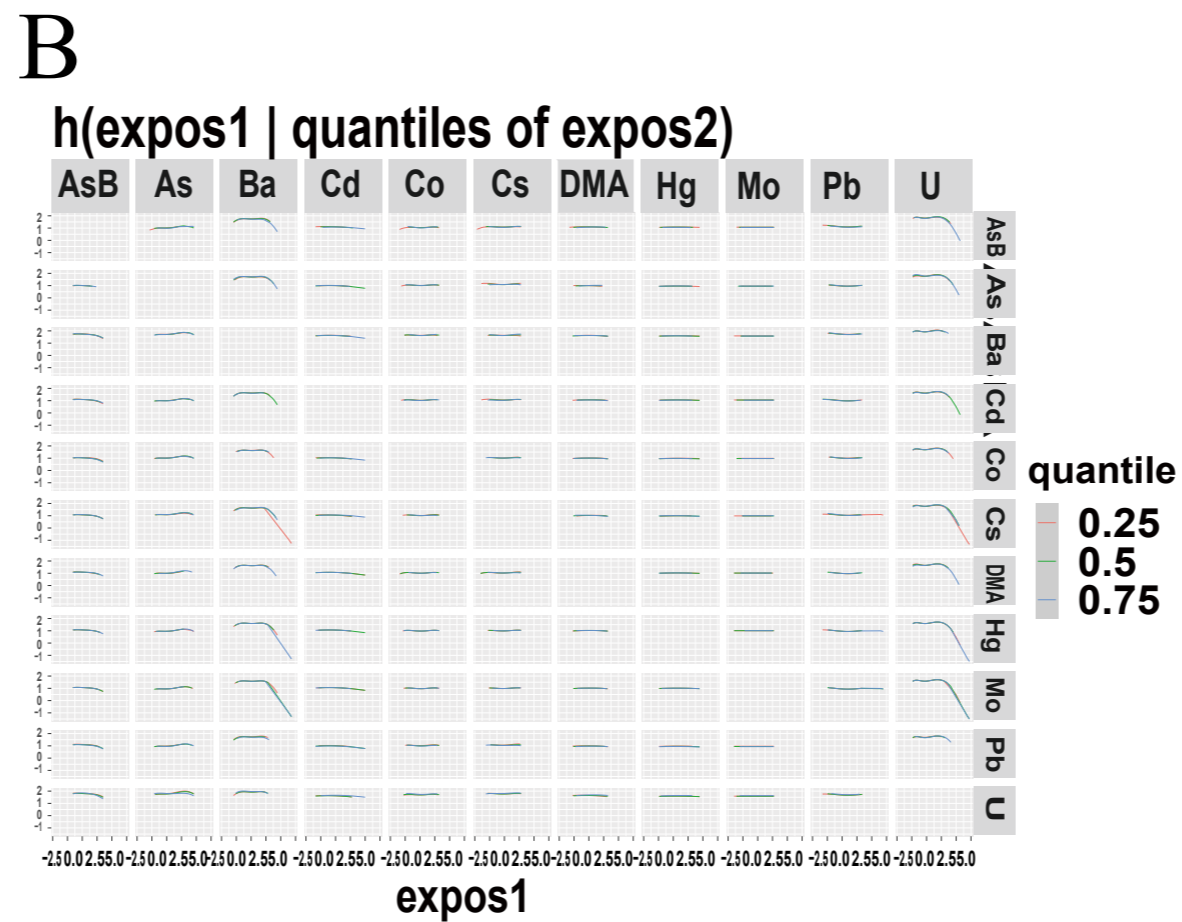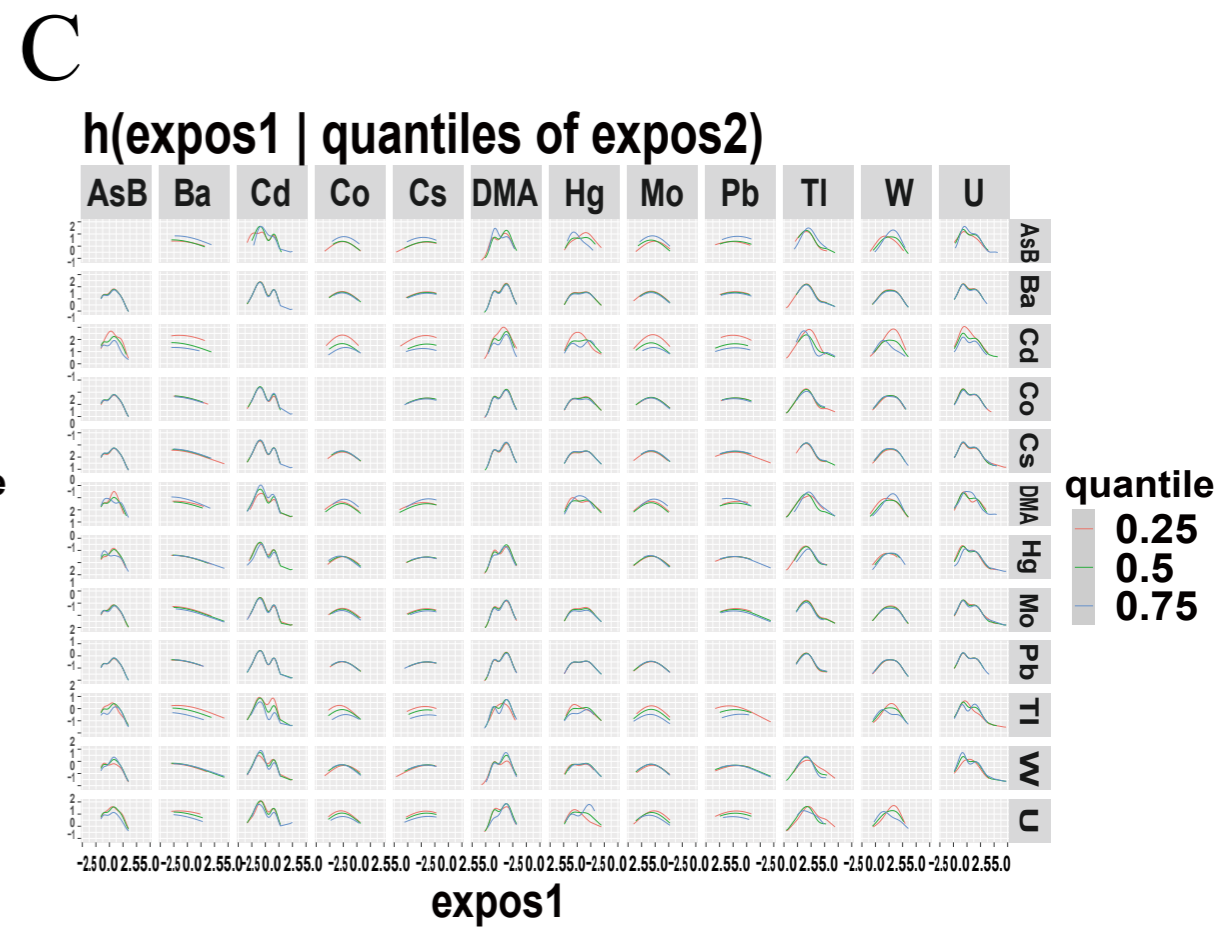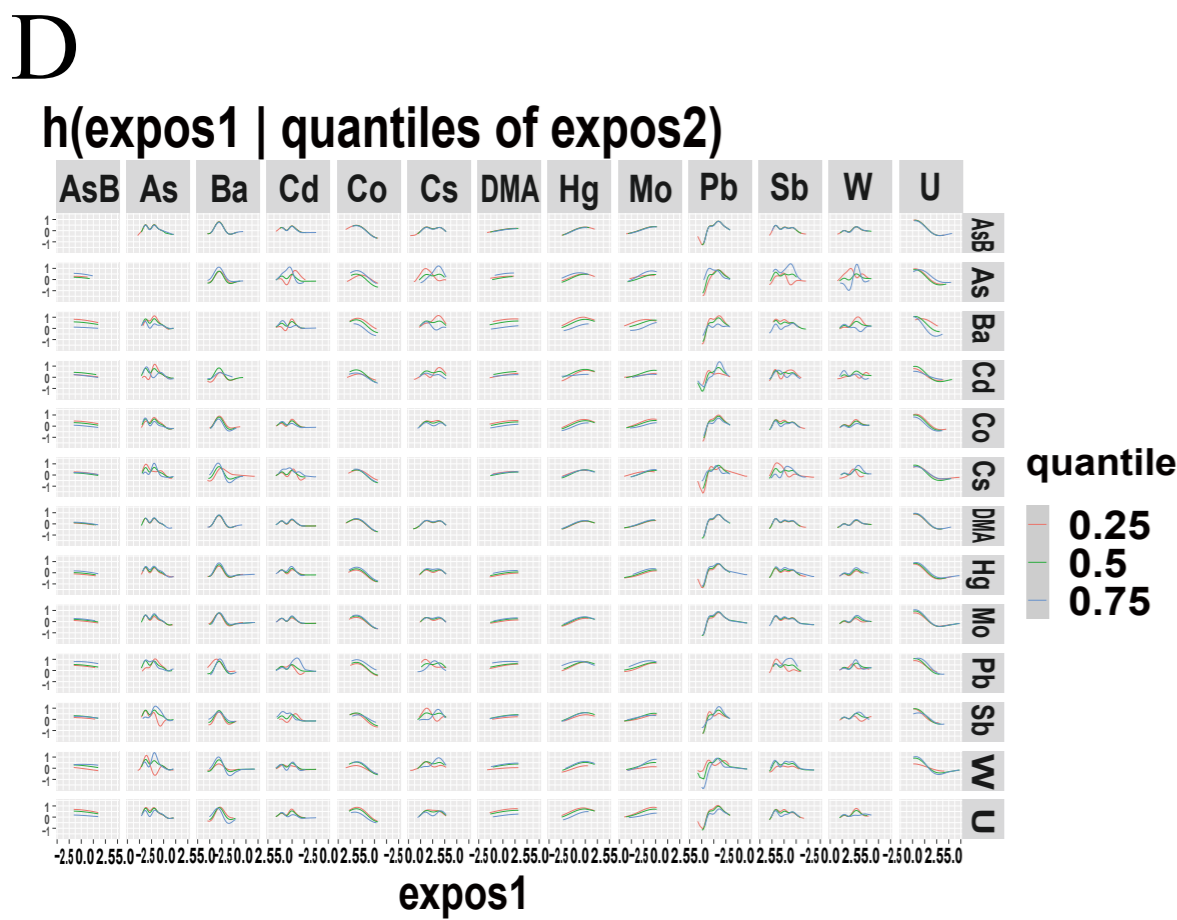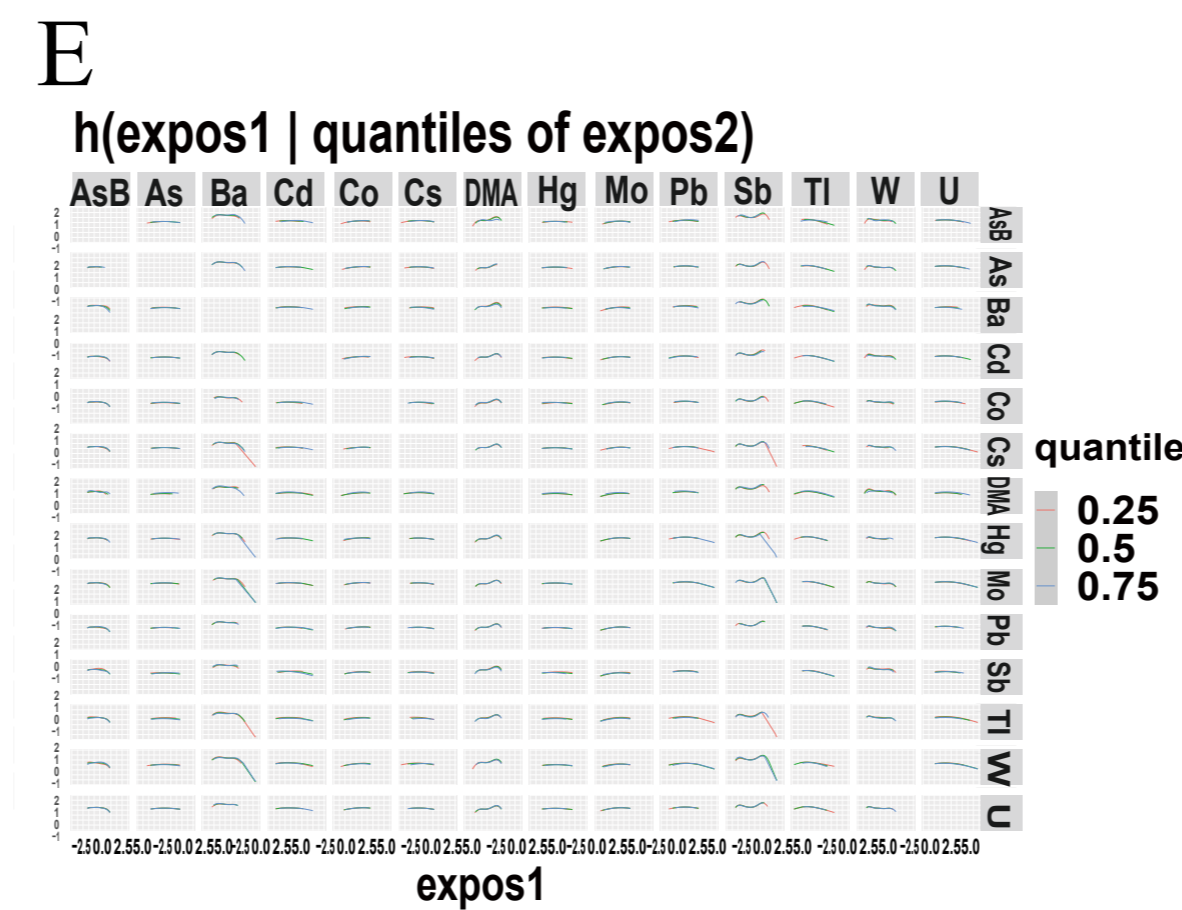

Supplement: Supplementary file 1 [file metabolites-14-00139-s001.zip › Figure S5.pdf]

A

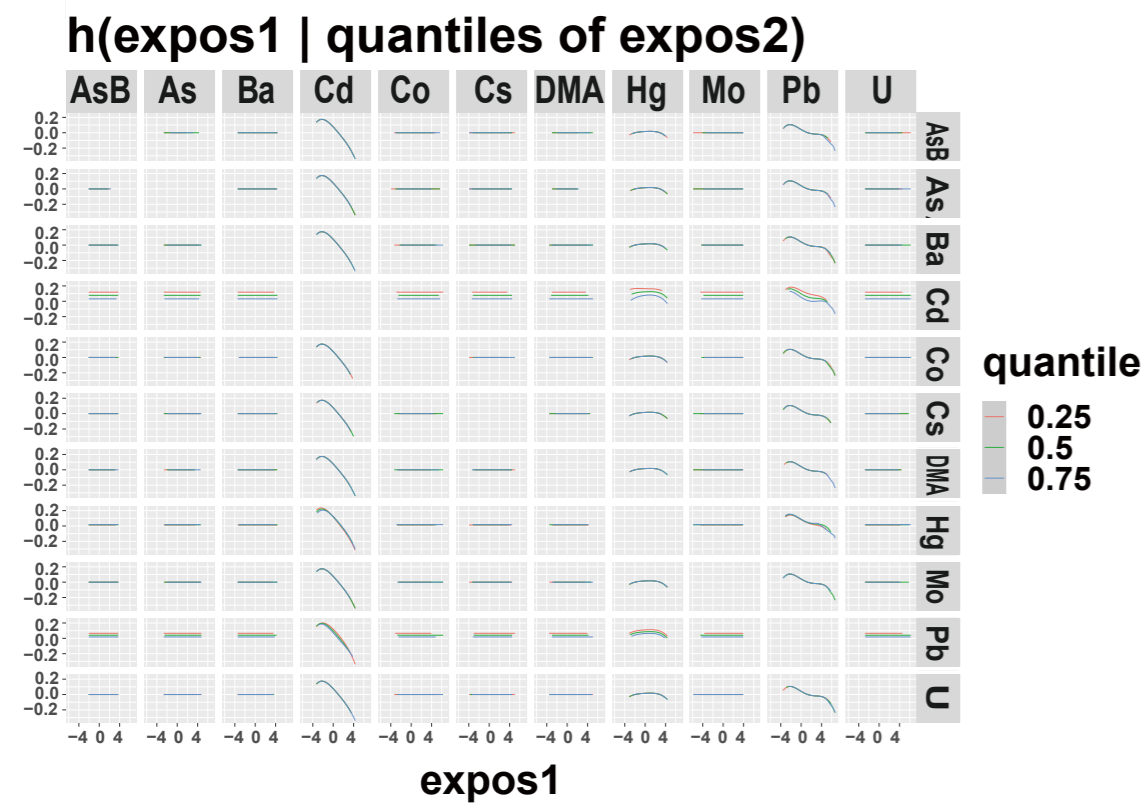

B

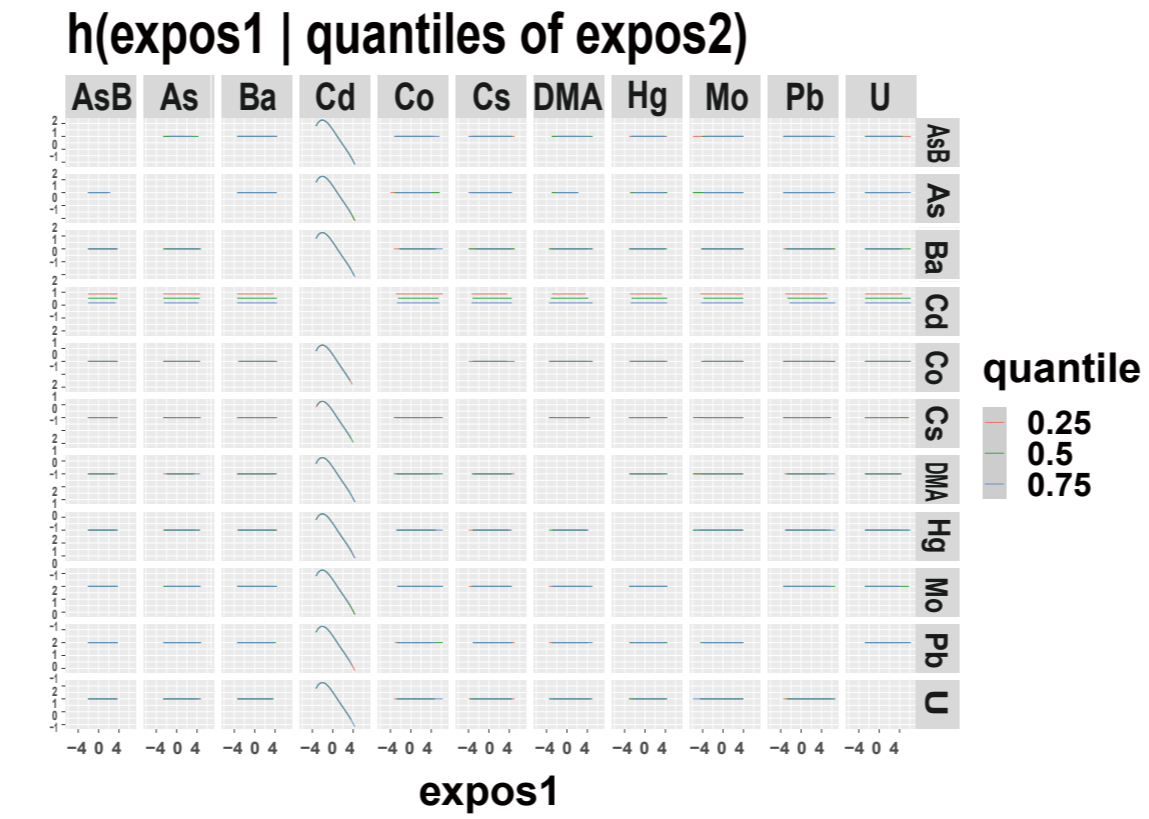

C

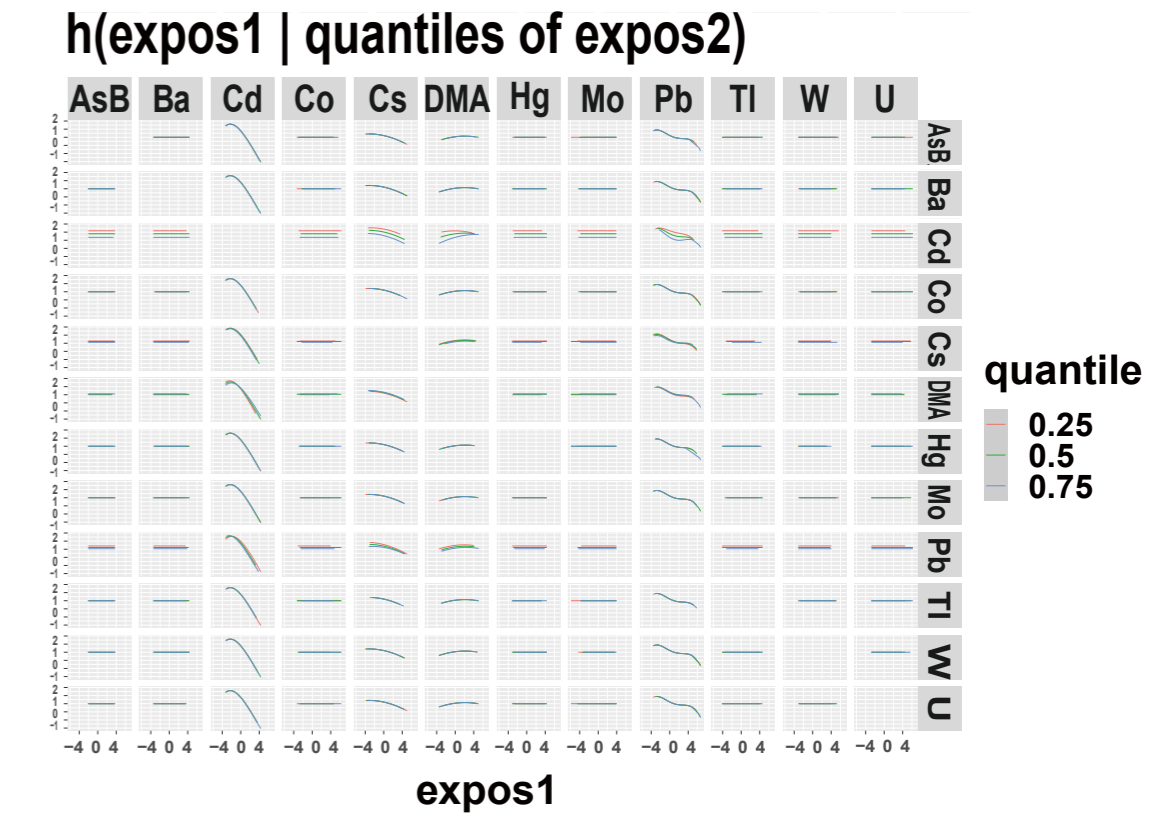

D

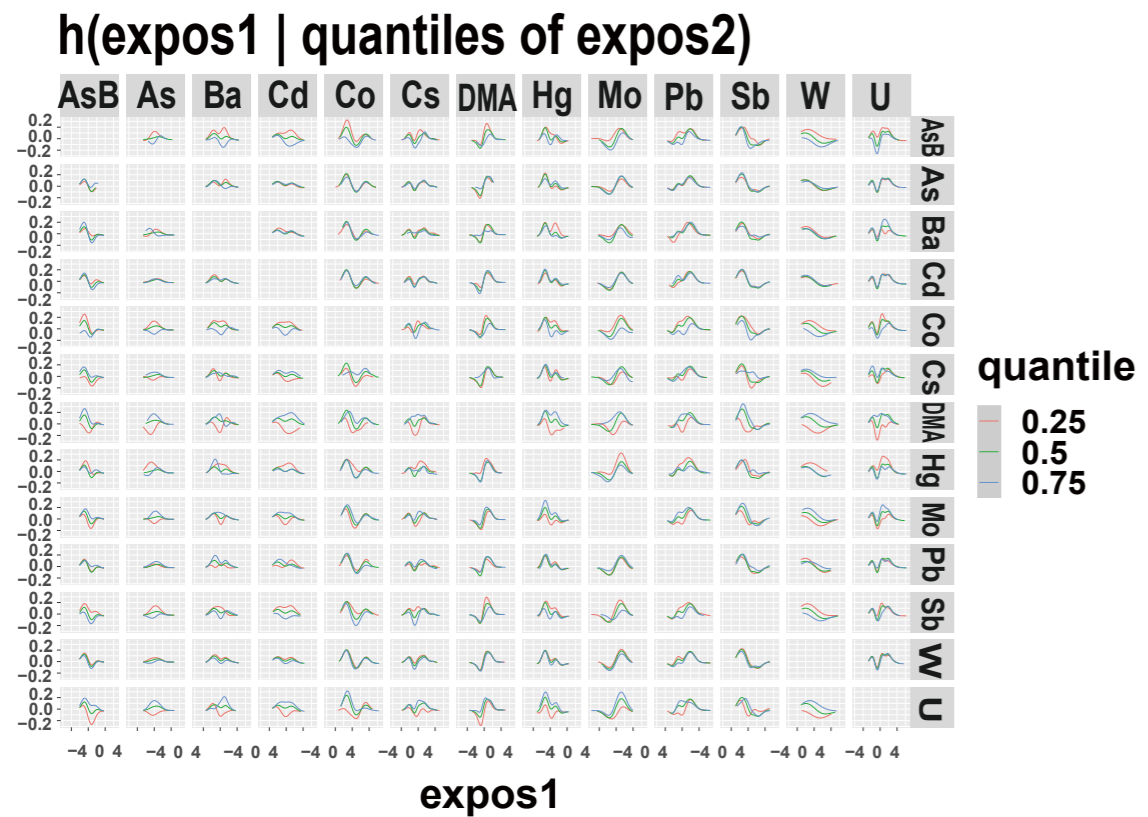

E

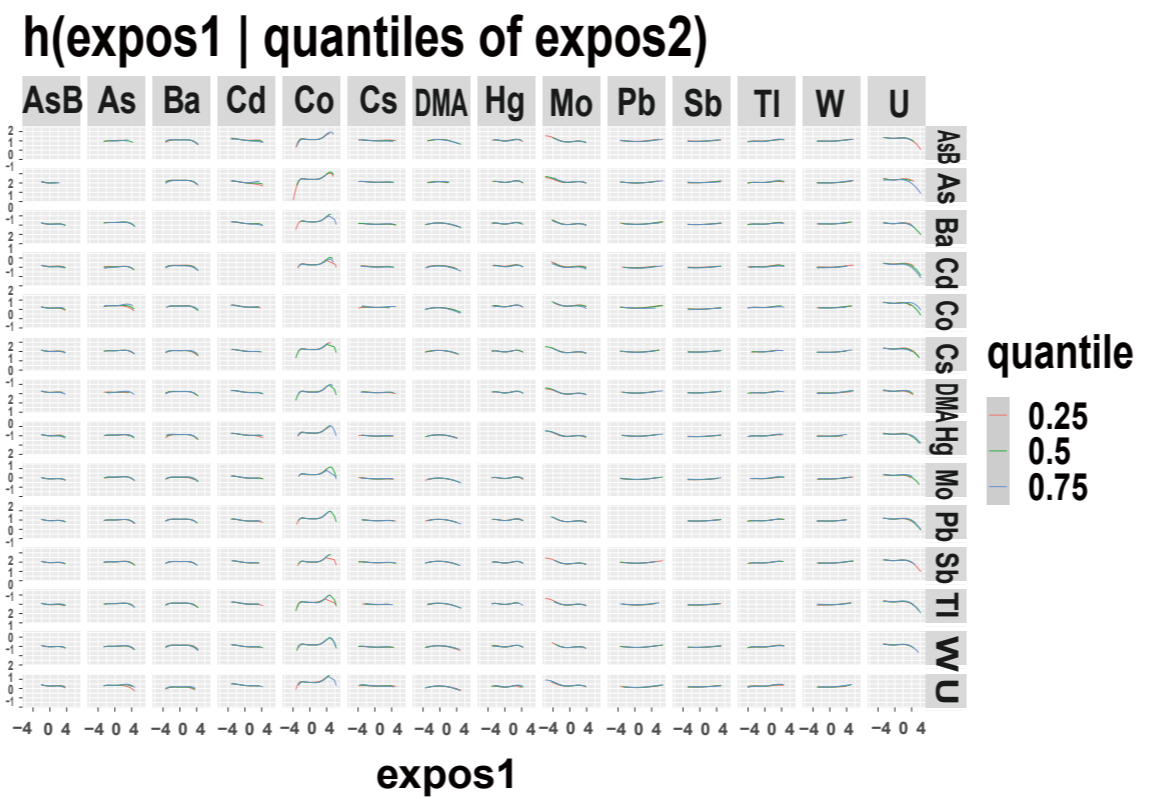

Supplement: Supplementary file 1 [file metabolites-14-00139-s001.zip › Figure S6.pdf]
